# Inhibition of the epithelial sodium channel (ENaC) by connexin 30 involves stimulation of clathrin-mediated endocytosis

**DOI:** 10.1016/j.jbc.2021.100404

**Published:** 2021-02-10

**Authors:** Alexandr V. Ilyaskin, Christoph Korbmacher, Alexei Diakov

**Affiliations:** Institut für Zelluläre und Molekulare Physiologie, Friedrich-Alexander-Universität Erlangen-Nürnberg (FAU), Erlangen, Germany

**Keywords:** epithelial sodium channel (ENaC), connexon (hemichannel), Nedd4-2, clathrin-mediated endocytosis, proteolytic channel activation, two-electrode voltage clamp, patch clamp, electrophysiology, oocyte, salt-sensitive hypertension, ANOVA, analysis of variance, AP-2, clathrin adaptor protein 2, ASDN, aldosterone-sensitive distal nephron, CD, collecting duct, cDNA, complementary DNA, CNT, connecting tubule, cRNA, complementary RNA, Cx, connexin, DCT2, late distal convoluted tubule, ENaC, epithelial sodium channel, GJ, gap-junctions, LJ, liquid junction, MTSET, (2-(trimethylammonium)ethyl) methanethiosulfonate bromide, Nedd4-2, neural precursor cell expressed developmentally downregulated protein 4-2, NMDG, N-methyl-D-glucamine, P_o_, channel open probability, PVDF, polyvinylidene fluoride, SDS-PAGE, sodium dodecyl sulphate-polyacrylamide gel electrophoresis, Sgk1, serum and glucocorticoid-induced kinase 1

## Abstract

Mice lacking connexin 30 (Cx30) display increased epithelial sodium channel (ENaC) activity in the distal nephron and develop salt-sensitive hypertension. This indicates a functional link between Cx30 and ENaC, which remains incompletely understood. Here, we explore the effect of Cx30 on ENaC function using the *Xenopus laevis* oocyte expression system. Coexpression of human Cx30 with human αβγENaC significantly reduced ENaC-mediated whole-cell currents. The size of the inhibitory effect on ENaC depended on the expression level of Cx30 and required Cx30 ion channel activity. ENaC inhibition by Cx30 was mainly due to reduced cell surface ENaC expression resulting from enhanced ENaC retrieval without discernible effects on proteolytic channel activation and single-channel properties. ENaC retrieval from the cell surface involves the interaction of the ubiquitin ligase Nedd4-2 with PPPxY-motifs in the C-termini of ENaC. Truncating the C- termini of β- or γENaC significantly reduced the inhibitory effect of Cx30 on ENaC. In contrast, mutating the prolines belonging to the PPPxY-motif in γENaC or coexpressing a dominant-negative *Xenopus* Nedd4 (xNedd4-CS) did not significantly alter ENaC inhibition by Cx30. Importantly, the inhibitory effect of Cx30 on ENaC was significantly reduced by Pitstop-2, an inhibitor of clathrin-mediated endocytosis, or by mutating putative clathrin adaptor protein 2 (AP-2) recognition motifs (YxxФ) in the C termini of β- or γ-ENaC. In conclusion, our findings suggest that Cx30 inhibits ENaC by promoting channel retrieval from the plasma membrane *via* clathrin-dependent endocytosis. Lack of this inhibition may contribute to increased ENaC activity and salt-sensitive hypertension in mice with Cx30 deficiency.

The epithelial sodium channel (ENaC) belongs to the ENaC/degenerin family of ion channels. ENaC is a heterotrimer consisting of three homologous subunits (αβγ) (1). Each subunit consists of two transmembrane domains connected by a large extracellular loop and has short intracellular N- and C-termini. In several sodium transporting epithelia, ENaC provides the rate-limiting step for apical sodium entry (2). In particular, ENaC is expressed in the apical membrane of principal cells in the aldosterone-sensitive distal nephron (ASDN). This nephron segment consists of the late distal convoluted tubule (DCT2), the connecting tubule (CNT), and the collecting duct (CD). The regulation of ENaC activity in the ASDN plays a key role in fine-tuning renal sodium excretion and hence in long-term control of arterial blood pressure (3, 4). ENaC activity in the ASDN critically depends on channel abundance in the apical membrane, which is determined by the balance between channel insertion into the plasma membrane and endocytic channel retrieval (4, 5, 6, 7). An essential hormonal regulator of ENaC activity in the ASDN is the mineralocorticoid aldosterone, but aldosterone-dependent and -independent ENaC regulation has been described in mouse distal nephron (8, 9, 10). The mechanisms involved in mediating the stimulatory effect of aldosterone on ENaC are highly complex and probably include increased forward trafficking of ENaC (11, 12) and reduced channel retrieval resulting in increased channel expression at the cell surface. There is good evidence that phosphorylation of the ubiquitin-protein ligase Nedd4-2 by serum and glucocorticoid-induced kinase 1 (Sgk1) mediates at least in part the stimulatory effect of aldosterone (13, 14). The C-termini of all three ENaC subunits contain proline-rich PPPxY (PY) motifs. Nedd4-2 interacts with PY-motifs *via* its WW-domains, promoting the ubiquitination, endocytosis, and proteasomal degradation of the channel (13, 15, 16, 17, 18, 19, 20, 21). Sgk1 is stimulated by aldosterone, and phosphorylation of Nedd4-2 by Sgk1 reduces the affinity of the PPPxY-motif to Nedd4-2, which attenuates channel retrieval from the cell surface, thereby increasing channel surface expression (5, 7, 20). The mechanisms by which Nedd4-2-dependent ubiquitination promotes ENaC internalization are not yet fully understood, but probably include clathrin-mediated endocytosis (22, 23, 24). It has been suggested that epsin, which has both ubiquitin-interacting and clathrin-binding motifs, may serve as a link between ubiquitinated ENaC subunits and the clathrin-dependent endocytic machinery (23). Additionally, clathrin-interacting proteins may directly bind ENaC and induce ENaC internalization. In particular, it has been shown that ENaC interacts with the μ-subunit of the clathrin adaptor protein 2 (AP-2) (23, 24). AP2 is a multisubunit complex formed of α, β, μ, and σ-subunits, which functions as a major hub in clathrin-mediated endocytosis binding clathrin itself and a plethora of accessory and cargo proteins (25, 26, 27). It has been shown that μAP2 initiates clathrin-mediated endocytosis by binding to a YxxΦ-motif present in numerous transmembrane proteins (26, 28, 29). Interestingly, C-termini of all three ENaC subunits contain YxxΦ-motifs, which partially overlap with the PPPxY motifs. It has been proposed that ENaC is regulated by two independent retrieval pathways, one involving Nedd4-2 and the other depending on μAP2 as key component (30). However, the functional role of the YxxΦ internalization motif in ENaC endocytosis remains to be elucidated.

In addition to aldosterone and several other hormones, numerous extracellular and intracellular factors including regulatory proteins can modulate ENaC activity in the ASDN. They include proteases, which activate ENaC by cleaving the channel at specific sites in the extracellular domains of the α- and γ-subunit. This releases inhibitory domains and probably causes a conformational change leading to channel activation (31, 32).

Interestingly, connexin 30 (Cx30) hemichannels have been suggested as putative ENaC modulators in the distal nephron (33, 34, 35, 36, 37). The canonical function of connexins is the formation of gap junctions (GJ) between adjacent cells (38, 39). Every GJ consists of two docked connexons providing electrical coupling and exchange of signaling molecules between adjacent cells. Connexin hemichannels may also play a physiological role in autocrine and/or paracrine signaling (40, 41, 42, 43, 44). It has been shown that various signaling molecules such as ATP, NAD^+^, glutathione, glutamate, PGE2, or polyamines can be released through connexin hemichannels (43, 44, 45). The connexin family consists of about 20 members in mammals (46). Among them, Cx26, Cx30, Cx30.3, Cx37, Cx43 are differentially expressed in various parts of the nephron (35, 37).

Importantly, Cx30 is expressed in the apical membrane of distal tubular epithelial cells in rodents suggesting that it may function there as a hemichannel (33). Apical Cx30 expression in the distal tubule was found to be upregulated by high-salt diet suggesting that Cx30 may be involved in the regulation of salt absorption in the distal nephron (33). Further studies supported this hypothesis. Cx30 knockout mice demonstrated impaired pressure natriuresis and salt-sensitive hypertension, which can be prevented by the ENaC inhibitor benzamil. This supports the hypothesis that Cx30 and ENaC may functionally interact in the distal nephron (34). Indeed, it has been shown that the open probability of ENaC and the number of active channels were increased in CCD of Cx30^−/−^ mice compared with wild-type mice (36). This may be due to a tonic inhibitory effect of Cx30 on ENaC possibly mediated by ATP release and purinergic signaling (47). Absence of this tonic inhibition in Cx30^−/−^ mice is thought to be responsible for increased ENaC activity. We hypothesized that additional molecular mechanisms may be involved in mediating the inhibitory effect of Cx30 on ENaC. The aim of this study was to investigate this further and to elucidate the underlying mechanisms of ENaC inhibition by Cx30 using the *Xenopus laevis* oocyte expression system.

## Results

### Functional expression of human Cx30 in *X. laevis* oocytes

**Figure 1:**
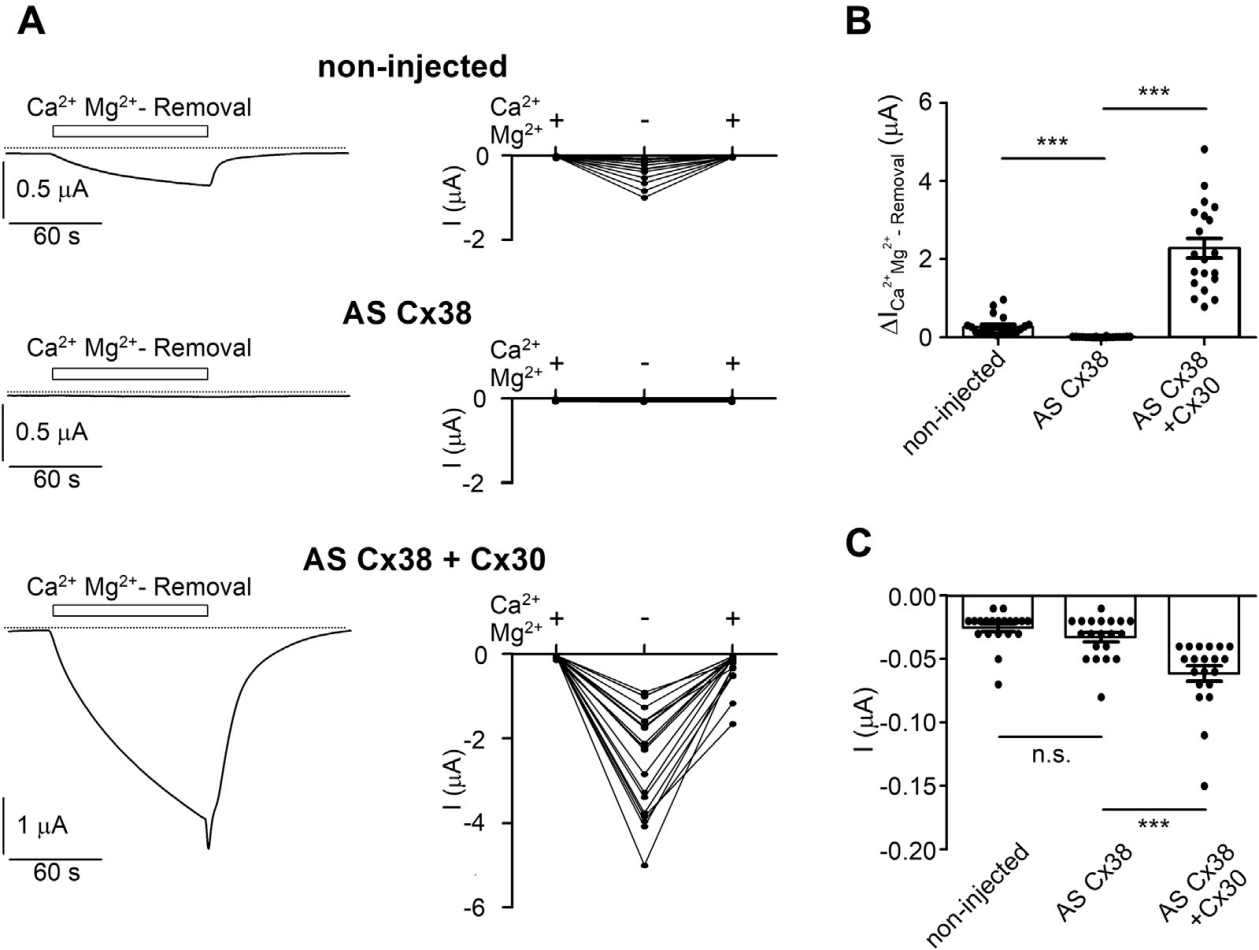
**Inhibition of endogenous Cx38 and functional expression of human Cx30 in *Xenopus laevis* oocytes**. *A*, *Left panels*, representative traces demonstrating the effect of divalent cation removal on whole-cell currents recorded at a holding potential of −60 mV in noninjected oocytes, oocytes injected with antisense oligoDNA against endogenous Cx38 (AS Cx38) or coinjected with AS Cx38 and human Cx30 cRNA (AS Cx38 + Cx30). Bath solution contained 1.8 mM Ca^2+^ and 1 mM Mg^2+^ or was nominally free of Ca^2+^ and Mg^2+^ and contained 2 mM EDTA for the time intervals (100 s) indicated by open bars (Ca^2+^Mg^2+^- Removal). *Dashed lines* indicate zero current level. *Right panels*, baseline inward currents in standard Ca^2+^and Mg^2+^-containing bath solution (left +), maximum inward currents in the absence of divalent cations (−), and minimum inward currents reached after reapplication of Ca^2+^ and Mg^2+^ ions (right +) from similar experiments as shown in *left panels* (*n* = 20; N = 2). *Lines* connect data points from individual oocytes. *B*, data from the experiments shown in (*A*) are summarized by subtracting the baseline current values in the presence of divalent cations from the corresponding maximal current value reached after Ca^2+^ and Mg^2+^-removal (ΔI_Ca_^_2+_^_Mg_^_2+_^_-Removal_). *C*, summary of baseline current values recorded at the beginning of each experiment in the presence of Ca^2+^ and Mg^2+^ in the bath solution. Mean ± SEM and data points for individual oocytes are shown; ∗∗∗*p* < 0.001; n.s., not significant; Kruskall–Wallis with Dunn’s post hoc test.

In a first set of experiments, we established the functional expression of human Cx30 in *X. laevis* oocytes. It has been reported that connexin hemichannels are inhibited by divalent cations (Ca^2+^, Mg^2+^) from the extracellular side (40, 48, 49). Therefore, we hypothesized that currents mediated by heterologously expressed Cx30 hemichannels may be revealed by removing divalent cations from the bath solution to disinhibit the channels. To test this, it was necessary to silence interfering endogenous connexin 38 (Cx38) hemichannels known to be expressed at the cell surface of *X. laevis* oocytes and to be activated by removal of extracellular Ca^2+^ and Mg^2+^ (50, 51, 52). As expected, concomitant removal of Ca^2+^ and Mg^2+^ from the bath solution reversibly stimulated inward whole-cell currents (ΔI_Ca_^_2+_^_Mg_^_2+_^_-Removal_) in noninjected oocytes confirming endogenous expression of functional Cx38 hemichannels (Fig. 1*A*, *upper panels*). It has been reported that endogenous expression of Cx38 in oocytes can be efficiently suppressed by antisense oligoDNA (AS Cx38) (50, 51) (see Experimental procedures). Using this established approach, we confirmed that in oocytes injected with AS Cx38, no inward currents were detectable upon Ca^2+^ and Mg^2+^ removal (Fig. 1*A*, *middle panels*). In contrast, in oocytes coinjected with AS Cx38 and cRNA for human Cx30, removal of Ca^2+^ and Mg^2+^ from the bath solution activated large inward currents consistent with the functional expression of Cx30 hemichannels at the cell surface (Fig. 1*A*, *lower panels*). Readdition of Ca^2+^ and Mg^2+^ rapidly returned the stimulated currents back to baseline. This inhibitory effect of Ca^2+^ and Mg^2+^ was usually preceded by a brief and variable inward current peak (Fig. 1*A*, *left lower panel*; Fig. 3*A*) probably due to a transient stimulation of Ca^2+^-activated chloride channels known to be endogenously expressed in oocytes (53). On average, inward currents elicited by Ca^2+^ and Mg^2+^ removal were not detectable in AS Cx38 injected control oocytes and were much higher in oocytes coinjected with AS Cx38 and cRNA for human Cx30 than those in noninjected oocytes expressing endogenous Cx38 (Fig. 1*B*). Thus, these initial experiments confirmed that endogenous Cx38 can be suppressed efficiently with AS Cx38. Moreover, they demonstrate that the oocyte system is suitable for heterologously expressing functional Cx30 hemichannels, which are present at the cell surface and can be activated by removing divalent cations from the bath solution.

**Figure 2:**
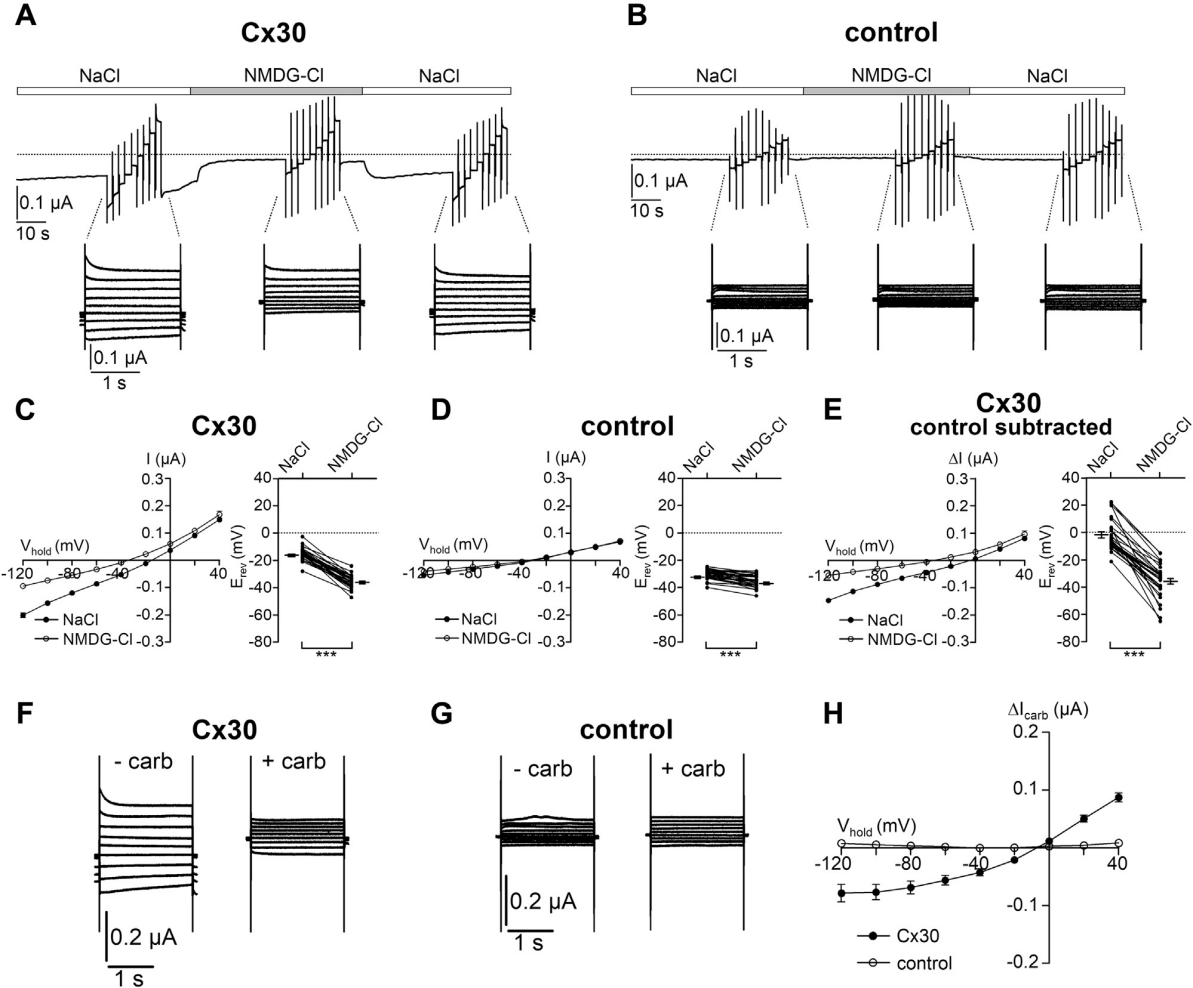
**Residual Cx30-mediated cation conductance in the presence of divalent cations (Ca**^**2+**^**, Mg**^**2+**^**) in the extracellular solution.***A* and *B*, representative whole-cell current traces recorded in a human Cx30 expressing oocyte (*A*) or in a control oocyte (*B*). Oocytes were superfused with standard bath solution (NaCl, *open bars*) or solution in which Na^+^ was replaced by NMDG^+^ (NMDG-Cl, *grey bar*). *Dashed lines* indicate zero current level. Continuous holding potential was −60 mV. Before, during, and after exposure to NMDG^+^ bath solution, voltage step protocols were performed with nine consecutive 2 s voltage steps in 20 mV increments starting with a hyperpolarizing pulse to −120 mV. *Insets* show overlays of resulting whole-cell current traces obtained at different holding potentials. *C* and *D*, *Left panels*, current data from the final 100 ms portion of each pulse were taken from similar experiments as shown in *A* and *B*, respectively, to construct corresponding average I/V curves. Mean and SEM (*n* = 34; N = 4) are shown. *Right panels*, reversal potentials obtained from the same experiments summarized in *left panels*. Individual data points and mean ± SEM are shown. *Lines* connect data points from individual oocytes. ∗∗∗*p* < 0.001; paired Student’s *t*-test. *E*, *Left panel*, average I/V plots corrected for endogenous oocyte currents using the data from the experiments summarized in *C* and *D*. The average whole-cell current values measured in control oocytes were subtracted from the corresponding individual whole-cell current values measured in oocytes from the same batch expressing Cx30. *Right panel*, reversal potentials obtained in the same experiments as summarized in the *left panel*. Individual data points and mean ± SEM are shown. ∗∗∗*p* < 0.001; paired Student’s *t*-test. *F* and *G*, representative overlays of whole-cell current traces resulting from similar voltage step protocols as described in *A* and *B* are shown from an oocyte expressing Cx30 (*F*) and from a control oocyte (*G*). For each oocyte current overlays are shown before (−carb) and 1 min after switching to a bath solution containing 1 mM carbenoxolone (+carb). *H*, current data from the final 100 ms portion of the pulses were taken from similar experiments as shown in *F* and *G*. Average *I/V* plots were constructed using carbenoxolone-sensitive current values (ΔI_carb_), which were calculated by subtracting the current values recorded in the presence of carbenoxolone (+carb) from the corresponding current values recorded in its absence (−carb). Mean and SEM (Cx30: *n* = 21; N = 2; control: *n* = 27; N = 2) are shown.

Interestingly, in the presence of divalent cations, baseline inward currents were significantly higher in oocytes coinjected with Cx30 cRNA and AS Cx38 than those in noninjected or AS Cx38-injected oocytes (Fig. 1*C*). This finding suggests that Cx30 hemichannels are not fully blocked in the presence of physiological concentrations of Ca^2+^ (1.8 mM) and Mg^2+^ (1 mM) in the extracellular solution but exhibit a small residual channel activity under these conditions. To investigate whether these baseline inward currents in Cx30 expressing oocytes are carried by Na^+^, we substituted Na^+^ in the bath solution with the large organic cation NMDG^+^. In Cx30 expressing oocytes (coinjected with AS Cx38), the baseline inward currents at a holding potential of −60 mV were significantly reduced by replacing Na^+^ with NMDG^+^ (Fig. 2*A*). This indicates that the current is mainly carried by Na^+^ as expected for a Cx30-mediated current. This is further supported by the leftward shift of the reversal potential from −15.3 ± 0.7 to −35.1 ± 0.8 mV (*p* < 0.001) observed upon switching from Na^+^ to NMDG^+^ in the bath solution (Fig. 2*C*). In contrast, in control oocytes without Cx30 (but injected with AS Cx38), replacing Na^+^ by NMDG^+^ had almost no effect on the magnitude of baseline inward currents (Fig. 2, *B* and *D*). Analysis of the reversal potential shifts upon switching from Na^+^ to NMDG^+^-containing solution in control oocytes revealed a marginal leftward shift from −30.4 ± 0.6 mV to −35.2 ± 0.7 mV (*p* < 0.001; Fig. 2*D*). The magnitude of this shift was significantly smaller than that observed in oocytes expressing Cx30 (−4.8 ± 0.6 in control oocytes *versus* −19.8 ± 0.9 mV in Cx30-expressing oocytes; *p* < 0.001). This indicates that the Na^+^ conductance of control oocytes is negligible. Thus, expression of Cx30 leads to a significant increase of a baseline cation conductance in oocytes. To estimate the cation permeability ratio (P_Na_:P_NMDG_) for Cx30, we had to correct for the contribution of endogenous oocyte channels to the observed currents. Therefore, we subtracted the average whole-cell current values measured in control oocytes at each holding potentials from the corresponding individual whole-cell current values measured in oocytes from the same batch expressing Cx30. From the resulting I/V curves, we determined the reversal potential shifts due to the replacement of Na^+^ in the bath solution by NMDG^+^ (Fig. 2*E*). From the average shift (−33.1 ± 2.2 mV), we estimated a permeability ratio P_Na_:P_NMDG_ of about 1.0 : 0.27. To the best of our knowledge, there are no data reported in the literature regarding the cation selectivity of Cx30 hemichannels. However, our estimate is in good agreement with previously published data on the ion selectivity of other connexin hemichannels, *e.g.*, Cx50 or Cx38 (40, 54, 55).

Next, we tested whether the Na^+^ conductance in Cx30-expressing oocytes can be blocked by the nonspecific connexin inhibitor carbenoxolone (56). Importantly, carbenoxolone-sensitive currents (ΔI_carb_) were detected in oocytes expressing Cx30 (Fig. 2, *F* and *H*) but not in control oocytes injected only with AS Cx38 (Fig. 2, *G* and *H*). This supports the conclusion that the increased baseline conductance in oocytes injected with Cx30 cRNA is due to Cx30 hemichannels expressed at the cell surface, which have a residual activity even in the presence of divalent cations in the bath solution.

Taken together, these experiments demonstrate that oocytes with silenced endogenous Cx38 are a suitable system for heterologously expressing functional human Cx30 hemichannels. These channels have a small baseline activity, mediate Na^+^ inward currents, and can be disinhibited by extracellular Ca^2+^ and Mg^2+^ removal. This latter maneuver can be used to assess functional Cx30 expression at the cell surface (see below). In all subsequent experiments, oocytes injected with cRNAs were routinely coinjected with AS Cx38 to suppress endogenous Cx38 expression.

### Coexpression of human Cx30 with human ENaC reduces amiloride-sensitive currents

**Figure 3:**
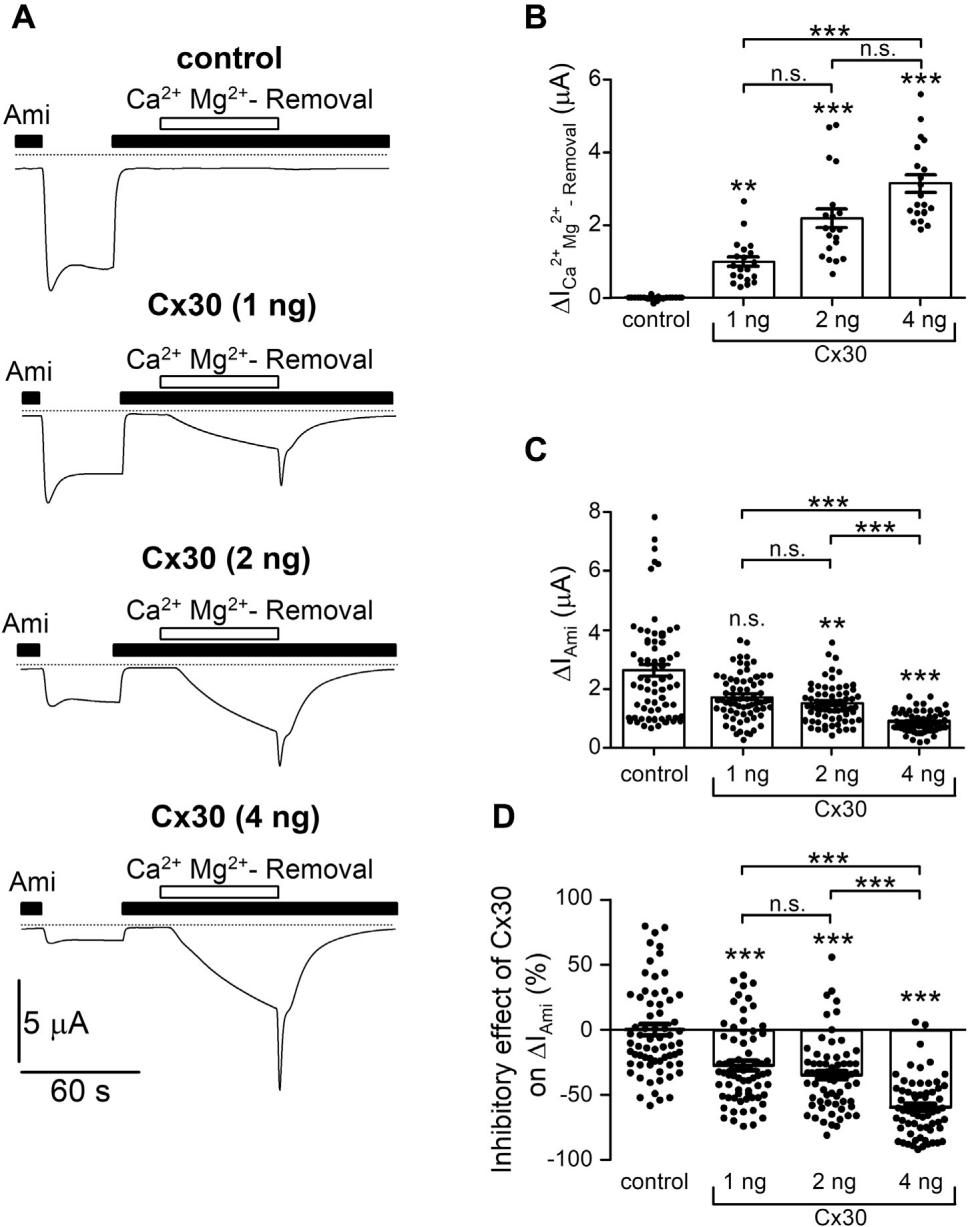
**Coexpression of Cx30 inhibits αβγENaC currents in a dose-dependent manner.***A*, representative whole-cell current traces recorded in a human αβγENaC-expressing control oocyte or in oocytes injected with both αβγENaC and different amounts of human Cx30 cRNA (1–4 ng/oocyte). Application of amiloride (Ami, 2 μM) and removal of divalent cations from the bath solution for 60 s (Ca^2+^Mg^2+^- Removal) are indicated by corresponding filled and open bars, respectively. *Dashed lines* indicate zero current level. *B*, ΔI_Ca_^_2+_^_Mg_^_2+_^_-Removal_ values were obtained from similar experiments as shown in *A* and calculated as described in Figure 1. Mean ± SEM and individual data points for each experiment are shown (*n* = 20, N = 5). *C*, ENaC-mediated amiloride-sensitive current values (ΔI_Ami_) were calculated from similar experiments as shown in A by subtracting the baseline current in the presence of amiloride from the current level reached after amiloride washout. Mean ± SEM and data points for individual oocytes are shown; (*n* = 68, N = 5). *D*, the relative inhibitory effect of Cx30 on ENaC was calculated according to the following equation: $\left(\frac{\Delta I_{Ami}}{\Delta I_{Ami}(mean,\;control)}-1\right)\times 100\%,$ where $\Delta I_{Ami}$ is the amiloride sensitive current of an individual oocyte coexpressing Cx30 and ENaC, whereas $\Delta I_{Ami}\left(mean,\;control\right)$ is the mean $\Delta I_{Ami}$ measured in control oocytes from the same batch but expressing ENaC alone (control). Original data are the same as in *C*. Mean ± SEM and data points for individual oocytes are shown; ∗∗*p* < 0.01; ∗∗∗*p* < 0.001; n.s., not significant; compared with control (markers above the columns) or to another comparison group as indicated; Kruskall–Wallis with Dunn’s post hoc test.

To investigate whether Cx30 can modify ENaC function, we expressed human αβγENaC in oocytes with or without coexpression of human Cx30. Amiloride-sensitive inward currents (ΔI_Ami_) and inward currents elicited by extracellular Ca^2+^ and Mg^2+^ removal (ΔI_Ca_^_2+_^_Mg_^_2+_^_-Removal_) were determined to assess ENaC and Cx30 function, respectively. As shown in Figure 3*A*, continuous whole-cell current recordings were performed at a holding potential of −60 mV and were started in the presence of amiloride (2 μM) to inhibit ENaC. Washout of amiloride revealed an ENaC-mediated sodium inward current (ΔI_Ami_). Reapplication of amiloride returned the current to its initial level. In control recordings from oocytes expressing ENaC without Cx30 (but coinjected with AS Cx38), subsequent removal of Ca^2+^ and Mg^2+^ had no detectable effect on the current level (Fig. 3*A*, control). In contrast, in oocytes coexpressing ENaC and Cx30, removal of Ca^2+^ and Mg^2+^ from the extracellular bath solution in the presence of amiloride increased the inward current by disinhibiting Cx30 hemichannels as described above. As expected, ΔI_Ca_^_2+_^_Mg_^_2+_^_-Removal_ increased when the amount of injected cRNA for Cx30 was increased from 1 ng to 2 ng or 4 ng (Fig. 3, *A* and *B*). This is not surprising, because it can be expected that increasing the amount of injected cRNA will increase channel expression. Importantly, increased expression of Cx30 resulted in progressive reduction of ΔI_Ami_ (Fig. 3, *A* and *C*). We calculated the relative inhibitory effect of Cx30 on ENaC (in %) for different amounts of injected Cx30 cRNA. The results are summarized in Figure 3*D* and demonstrate that Cx30 significantly downregulates ENaC in a dose-dependent manner. A significant inhibitory effect of Cx30 on ENaC was consistently observed in 33 batches of oocytes coinjected with 2 ng of Cx30 cRNA per oocyte and 0.1 ng of cRNA for each ENaC subunit. In these experiments, the relative inhibitory effect of Cx30 on ENaC averaged 54 ± 3% (N = 33, *n* = 496).

In conclusion, these data demonstrate that coexpression of Cx30 has a robust inhibitory effect on ENaC currents. The inhibitory effect of Cx30 increases with increasing expression of Cx30.

### Inhibitory effect of Cx30 on ENaC requires ion channel activity of Cx30

**Figure 4:**
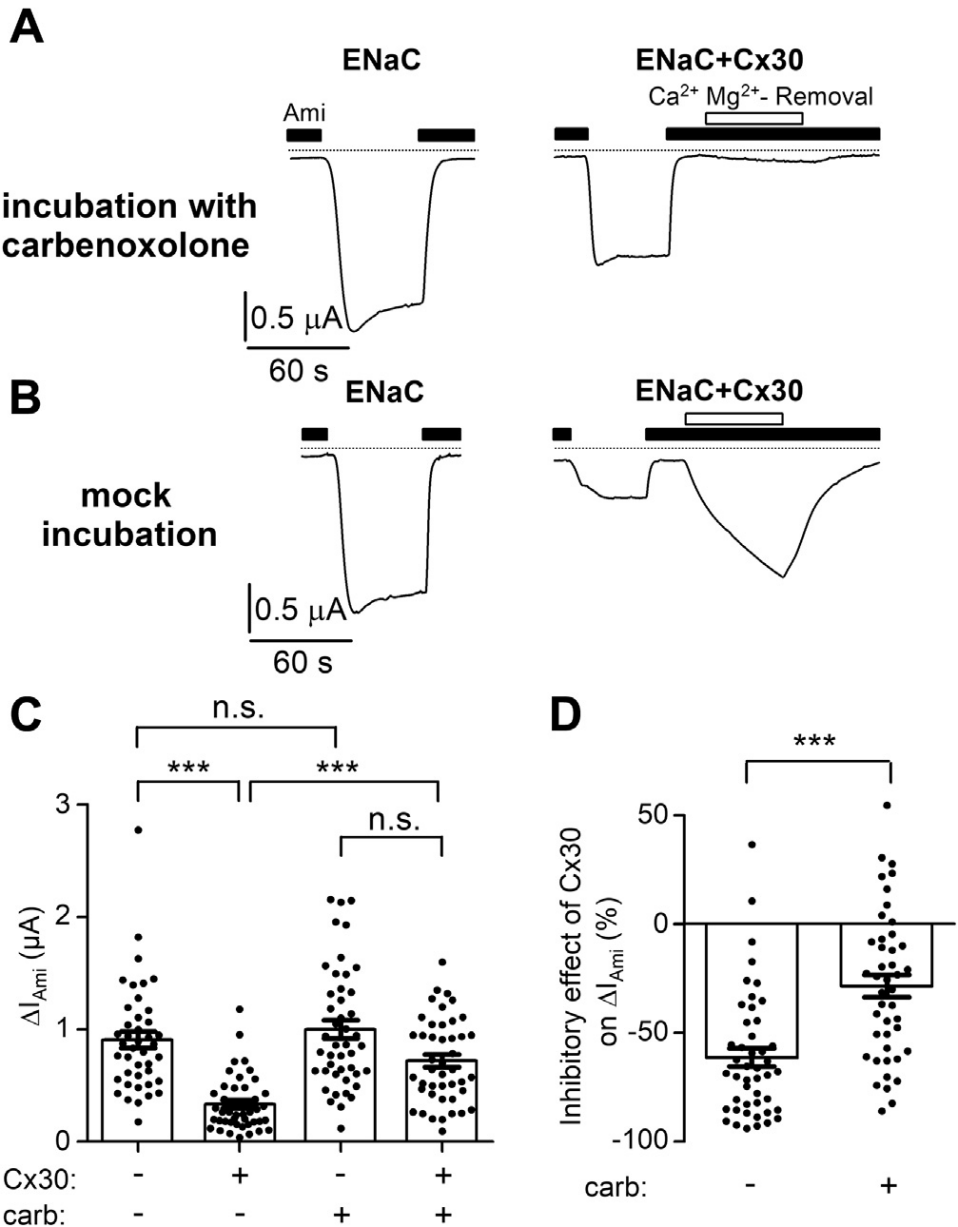
**Carbenoxolone significantly reduces the inhibitory effect of Cx30 on ENaC.***A* and *B*, representative whole-cell current traces recorded in oocytes expressing ENaC without (*left traces*) or with Cx30 (*right traces*). After cRNA injection oocytes were incubated for 48 h in incubation solution with carbenoxolone (100 μM, *A*) or without (mock incubation, *B*). Carbenoxolone was absent from the bath solution during the whole-cell current recordings. Application of amiloride (Ami, 2 μM) and removal of divalent cations from the bath solution for 60 s (Ca^2+^ Mg^2+^-Removal) are indicated by corresponding bars. *Dashed lines* indicate zero current level. *C*, ΔI_Ami_ values from similar experiments as shown in the representative traces in *A* and *B*. Mean ± SEM and data points for individual oocytes are shown; ∗∗∗*p* < 0.001; n.s., not significant; Kruskall–Wallis with Dunn’s post hoc test (*n* = 44, N = 4). *D*, relative inhibitory effect of Cx30 on ENaC calculated from the data shown in C essentially as described in Figure 3. Mean ± SEM and data points for individual oocytes are shown; ∗∗∗*p* < 0.001; Mann–Whitney test.

As described above, Cx30 expressing oocytes exhibit a carbenoxolone-sensitive conductance in the presence of physiological concentrations of Ca^2+^ and Mg^2+^ in the bath solution (Fig. 2). To test whether this channel activity is relevant for the inhibitory effect of Cx30 on ENaC, we incubated oocytes for 48 h after cRNA injection with the Cx30 blocker carbenoxolone (100 μM). In oocytes expressing ENaC and Cx30, preincubation with carbenoxolone prevented the stimulation of Cx30 mediated inward currents upon removal of Ca^2+^ and Mg^2+^ from the extracellular solution (Fig. 4*A*, *right trace*). Interestingly, this inhibitory effect of carbenoxolone preincubation was observed in the absence of carbenoxolone during the current measurements. This may be due to its intracellular accumulation and/or incomplete washout. In contrast, a stimulatory response to Ca^2+^ and Mg^2+^ removal was readily observed in matched ENaC and Cx30 coexpressing oocytes not treated with carbenoxolone (Fig. 4*B*, *right trace*). Importantly, average ENaC currents were more than twofold larger in carbenoxolone treated oocytes coexpressing ENaC and Cx30 than those in matched oocytes coexpressing ENaC and Cx30 but without carbenoxolone treatment (Fig. 4*C*). Without coexpression of Cx30, carbenoxolone had no significant effect on ENaC-mediated currents in ENaC expressing oocytes from the same batch (Fig. 4, *A* and *B*, *left traces*, and Fig. 4*C*). Thus, inhibition of Cx30 by incubation of oocytes in the presence of carbenoxolone significantly reduced the inhibitory effect of Cx30 on ENaC (Fig. 4*D*). In an additional set of experiments, we demonstrated that in oocytes coexpressing ENaC and Cx30, acute application of carbenoxolone (1 mM) for 1 min had no apparent effect on ΔI_Ami_. Compared with ΔI_Ami_ in oocytes expressing ENaC alone, ΔI_Ami_ was reduced in oocytes coexpressing ENaC and Cx30 by 70 ± 4% before and by 69 ± 5% after application of carbenoxolone (n.s.; N = 1, *n* = 10). Thus, acute application of carbenoxolone had no detectable effect on ENaC inhibition by Cx30 coexpression. These findings indicate that a prolonged ion channel function of Cx30 is needed to cause the observed inhibitory effect of Cx30 coexpression on ENaC and that short-term inhibition of Cx30 by carbenoxolone cannot reverse this effect.

**Figure 5:**
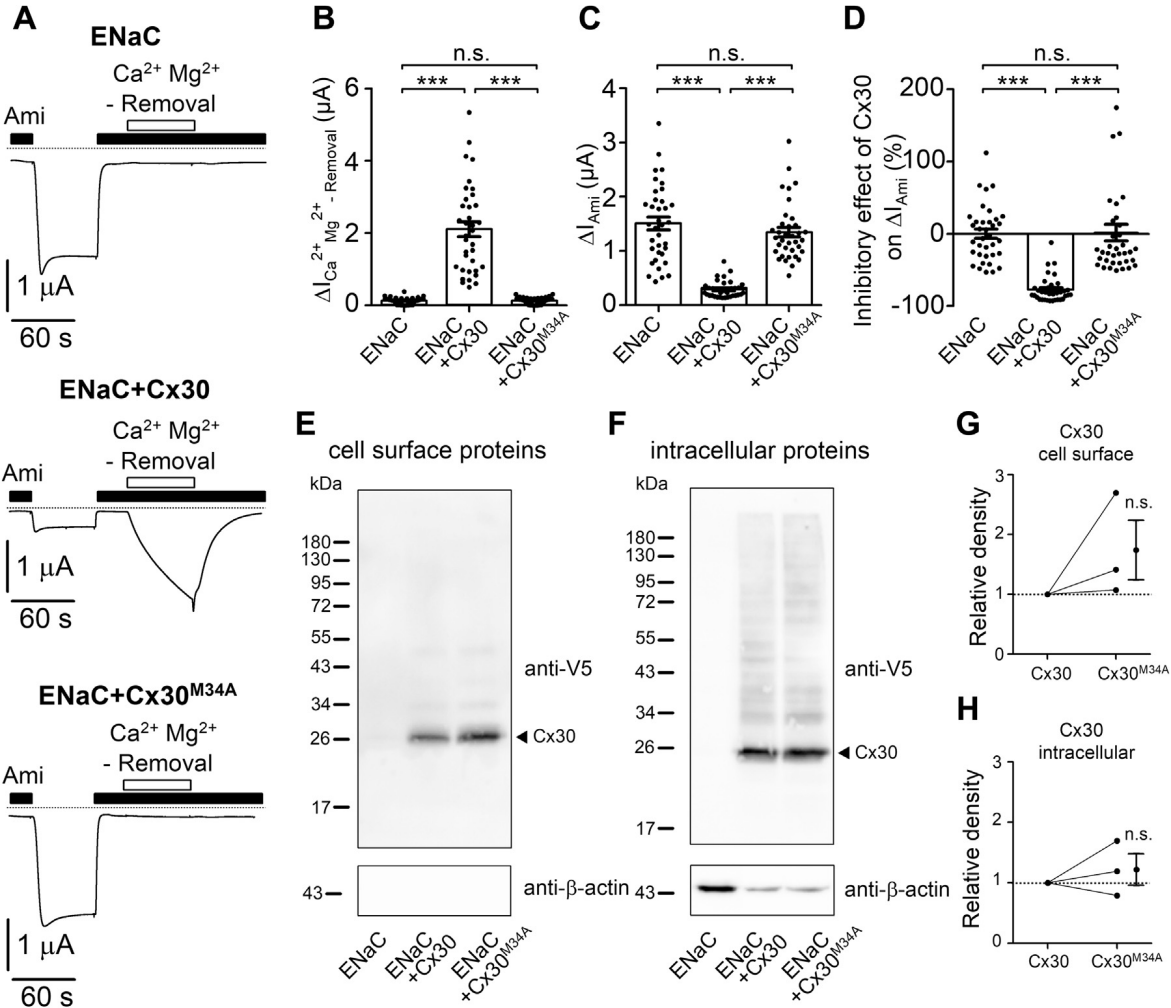
**Mutant Cx30 (M34A) with no detectable ion channel activity did not inhibit ENaC.***A*, representative whole-cell current traces recorded in oocytes expressing ENaC only (*upper trace*), coexpressing ENaC with C-terminally V5-tagged wild-type Cx30 (ENaC + Cx30, *middle trace*) or Cx30 with single point mutation M34A (ENaC + Cx30^M34A^, *lower trace*). Application of amiloride (Ami, 2 μM) and removal of divalent cations from the bath solution for 60 s (Ca^2+^ Mg^2+^-Removal) are indicated by *corresponding bars*. *Dashed lines* indicate zero current level. *B*, ΔI_Ca_^_2+_^_Mg_^_2+_^_-Removal_ values were obtained from similar experiments as shown in *A* and calculated as described in Figure 1. Mean ± SEM and individual data points for each experiment are shown; ∗∗∗*p* < 0.001; n.s., not significant; one-way ANOVA with Bonferroni post hoc test (*n* = 36, N = 3). *C*, ΔI_Ami_ were calculated from similar experiments as shown in A as described in Figure 3. Mean ± SEM and data points for individual oocytes are shown; ∗∗∗*p* < 0.001; n.s., not significant; Kruskall–Wallis with Dunn’s post hoc test. *D*, relative inhibitory effect of Cx30 on ENaC calculated from the data shown in *C* essentially as described in Figure 3. Mean ± SEM and data points for individual oocytes are shown; ∗∗∗*p* < 0.001; n.s., not significant; Kruskall–Wallis with Dunn’s post hoc test. *E* and *F*, representative western blots showing cell surface (*E*) or intracellular (*F*) expression of C-terminally V5-tagged wild-type Cx30 and mutant Cx30^M34A^ in oocytes from the same batch as used in *A*. No specific signal was detected with the anti-V5 antibody in oocytes expressing ENaC alone. To validate separation of cell surface proteins from intracellular proteins, blots were stripped and reprobed using an antibody against β-actin. *G* and *H*, densitometric evaluation of Cx30 and Cx30^M34A^ expression from three similar blots as shown in *E* and *F*. In each blot the density value of the Cx30^M34A^ band was normalized to that of the Cx30 band. *Lines* connect data points obtained in the same experiment, and mean ± SEM are shown; n.s. not significant; one sample Wilcoxon signed-rank test (N = 3).

Carbenoxolone is a rather nonspecific drug and may have additional side effects besides blocking Cx30. Therefore, we established an alternative approach to silence the ion channel activity of Cx30. It has been shown for Cx26, a close homologue of Cx30, that introducing a point mutation into the pore-forming transmembrane domain 1 (M34T or M34A) renders Cx26 inactive by constricting the channel pore (57, 58). Interestingly, the M34T mutation of Cx26 was found to be associated with hereditary deafness in humans (59, 60). We hypothesized that due to the high degree of sequence similarity between Cx26 and Cx30 (approx. 77%), a homologous M34A substitution in Cx30 would also result in nonfunctional channels. Indeed, as shown in Figure 5, *A* and *B*, divalent cation removal from the bath solution did not elicit an inward current response in oocytes expressing ENaC together with the mutant Cx30 (ENaC+Cx30^M34A^). This lack of response to divalent cation removal is similar to that in control oocytes expressing ENaC alone and confirms the loss-of-function effect of the M34A mutation on Cx30 ion channel function. In contrast, in matched oocytes coexpressing ENaC with wild-type Cx30 (ENaC+Cx30), the usual inward current response to divalent cation removal was detected. Importantly, in contrast to Cx30, Cx30^M34A^ did not significantly reduce ΔI_Ami_ (Fig. 5, *C* and *D*). Western blot analysis revealed that intracellular and cell surface expression of Cx30^M34A^ was similar to that of Cx30 (Fig. 5, *E*–*H*). Thus, the M34A mutation effectively suppresses ion channel function of Cx30 without compromising its level of protein expression or its localization at the cell surface. Taken together these findings support our conclusion that the ion channel function of Cx30 is essential for its inhibitory effect on ENaC. They also argue against the possibility that Cx30 inhibits ENaC simply by competing for the transcription or expression machinery of the oocyte.

**Figure 6:**
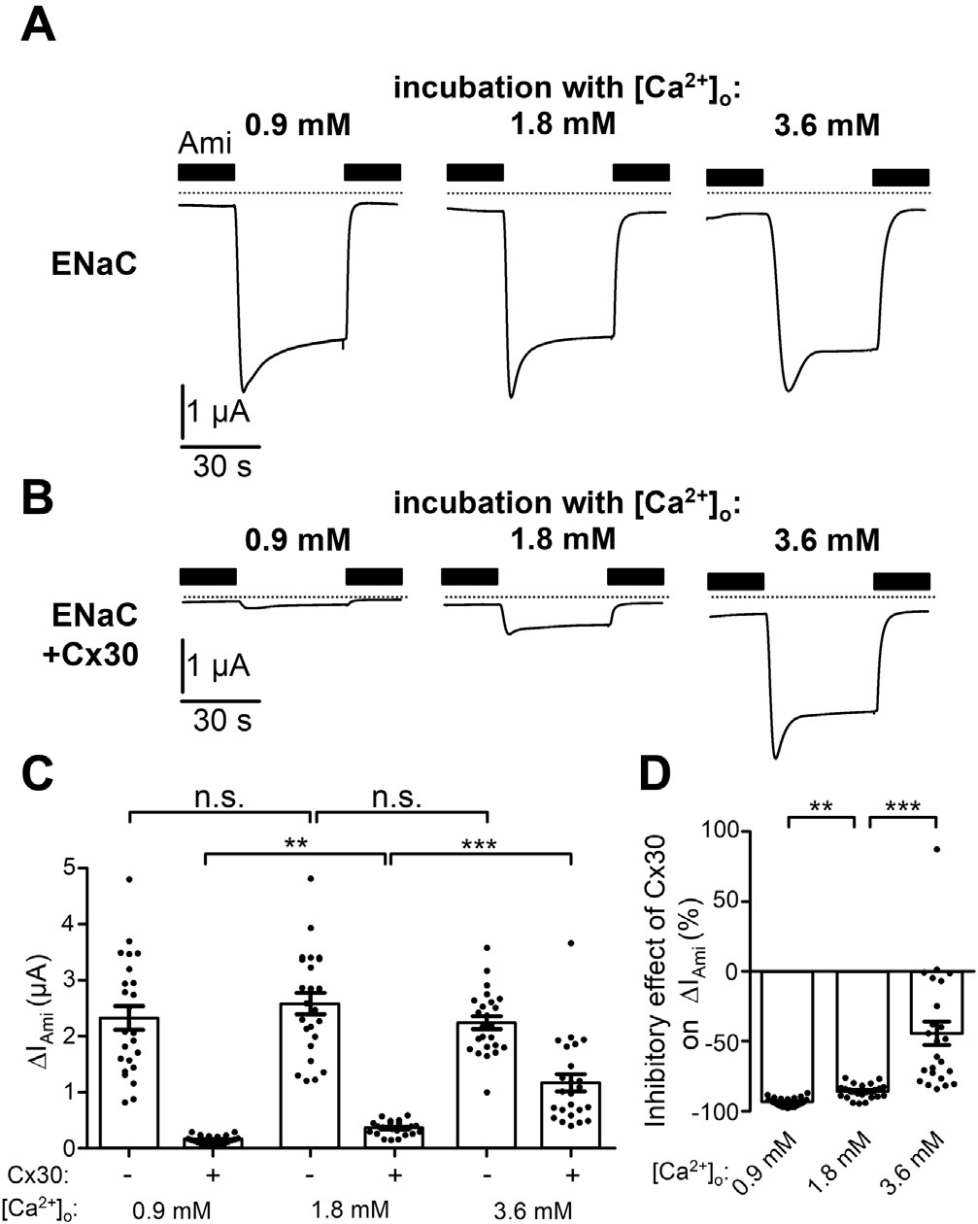
**Extracellular Ca**^**2+**^**modulates the inhibitory effect of Cx30 on ENaC.***A* and *B*, representative whole-cell current traces recorded in oocytes expressing ENaC only (*A*) or coexpressing ENaC with Cx30 (*B*) incubated for 48 h in solution containing the indicated concentration of Ca^2+^ ([Ca^2+^]_o_ = 0.9, 1.8 or 3.6 mM). Application of amiloride (Ami, 2 μM) is indicated by filled bars. *Dashed lines* indicate zero current level. *C*, ΔI_Ami_ values from similar experiments as shown in the representative traces (*A* and *B*). Mean ± SEM and data points for individual oocytes are shown; ∗∗∗*p* < 0.001; ∗∗*p* < 0.01; n.s., not significant; Kruskall–Wallis with Dunn’s post hoc test (*n* = 24, N = 2). *D*, relative inhibitory effect of Cx30 on ENaC calculated from the data shown in (*C*) essentially as described in Figure 3. Mean ± SEM and data points for individual oocytes are shown; ∗∗∗*p* < 0.001; ∗∗*p* < 0.01; Kruskall–Wallis with Dunn’s post hoc test.

**Figure 7:**
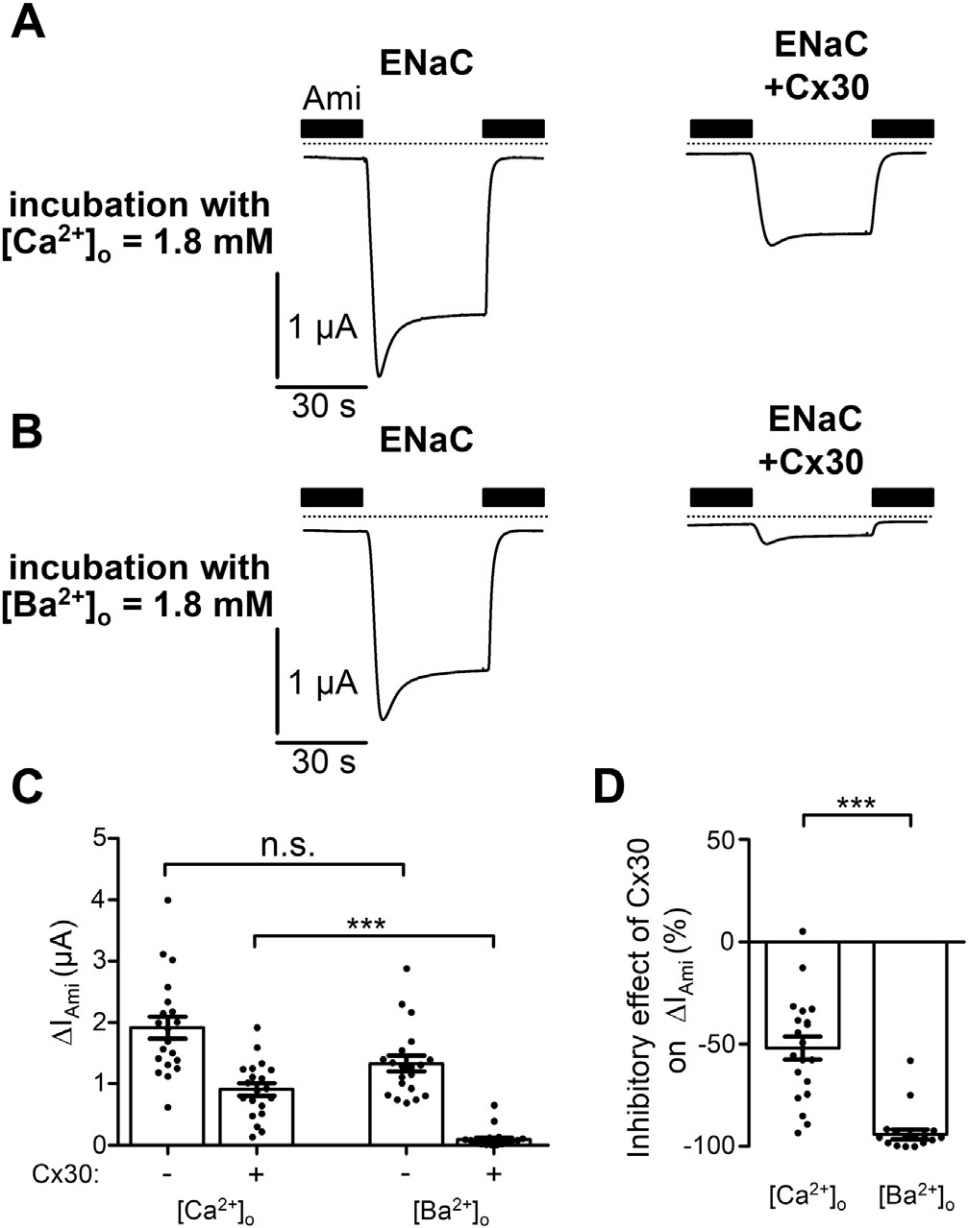
**Replacing extracellular Ca**^**2+**^**with Ba**^**2+**^**enhances Cx30-mediated inhibition of ENaC.***A* and *B*, representative whole-cell current traces recorded in oocytes expressing ENaC alone (*left panels*) or coexpressing ENaC with Cx30 (*right panels*) incubated for 48 h in standard incubation solution containing 1.8 mM Ca^2+^ ([Ca^2+^]_o_ = 1.8 mM; *A*) or in incubation solution in which 1.8 mM Ca^2+^ was replaced with 1.8 mM Ba^2+^ ([Ba^2+^]_o_ = 1.8 mM; *B*). Application of amiloride (Ami, 2 μM) is indicated by filled bars. *Dashed lines* indicate zero current level. *C*, ΔI_Ami_ values from similar experiments as shown in the representative traces *A* and *B*. Mean ± SEM and data points for individual oocytes are shown; ∗∗∗*p* < 0.001; n.s., not significant; Kruskall–Wallis with Dunn’s post hoc test (*n* = 20, N = 2). *D*, relative inhibitory effect of Cx30 on ENaC calculated from the data shown in *C* essentially as described in Figure 3. Mean ± SEM and data points for individual oocytes are shown; ∗∗∗*p* < 0.001; Mann–Whitney test.

It has been reported that decreasing or increasing the extracellular Ca^2+^ concentration enhances or reduces ion permeation through Cx30 hemichannels, respectively (61). Therefore, we hypothesized that the inhibitory effect of Cx30 on ENaC may be modified by varying the extracellular Ca^2+^ concentration. To test this, we incubated oocytes expressing ENaC with or without Cx30 for 48 h after cRNA injection in ND9 with a standard (1.8 mM), decreased (0.9 mM), or increased (3.6 mM) Ca^2+^ concentration. As shown in Figure 6, *A* and *C*, varying the Ca^2+^ concentration in the incubation medium had no effect on ΔI_Ami_ in oocytes expressing ENaC without Cx30. In contrast, the inhibitory effect of Cx30 coexpression on ΔI_Ami_ was slightly more pronounced with 0.9 mM and substantially attenuated with 3.6 mM extracellular Ca^2+^ compared with its effect under control conditions with 1.8 mM extracellular Ca^2+^ (Fig. 6, *B* and *C*). Thus, there appears to be an inverse relationship between the Ca^2+^ concentration of the incubation solution and the relative inhibitory effect of Cx30 on ENaC (Fig. 6*D*). As stated above, Cx30 hemichannels are inhibited by extracellular Ca^2+^. Therefore, these finding suggests that the degree of ENaC inhibition by Cx30 correlates with the ion channel activity of Cx30, which is modulated by the extracellular Ca^2+^ concentration. However, the inhibitory effect of extracellular Ca^2+^ on Cx30 does not rule out the possibility that Cx30 hemichannels mediate Ca^2+^ influx and modulate intracellular Ca^2+^ signaling. To investigate whether the inhibitory effect of coexpressed Cx30 on ENaC depends on the presence of extracellular Ca^2+^, we substituted Ca^2+^ in the incubation solution by Ba^2+^. In oocytes expressing ENaC without Cx30, replacement of Ca^2+^ by Ba^2+^ had no significant effect on ΔI_Ami_. Importantly, in oocytes incubated in the presence of Ba^2+^ instead of Ca^2+^, the inhibitory effect of Cx30 coexpression on ΔI_Ami_ was not only preserved but even enhanced (Fig. 7, *A*–*D*). Thus, extracellular Ca^2+^ is not necessary for mediating the inhibitory effect of Cx30 on ENaC.

### Cx30 reduces surface expression of ENaC

**Figure 8:**
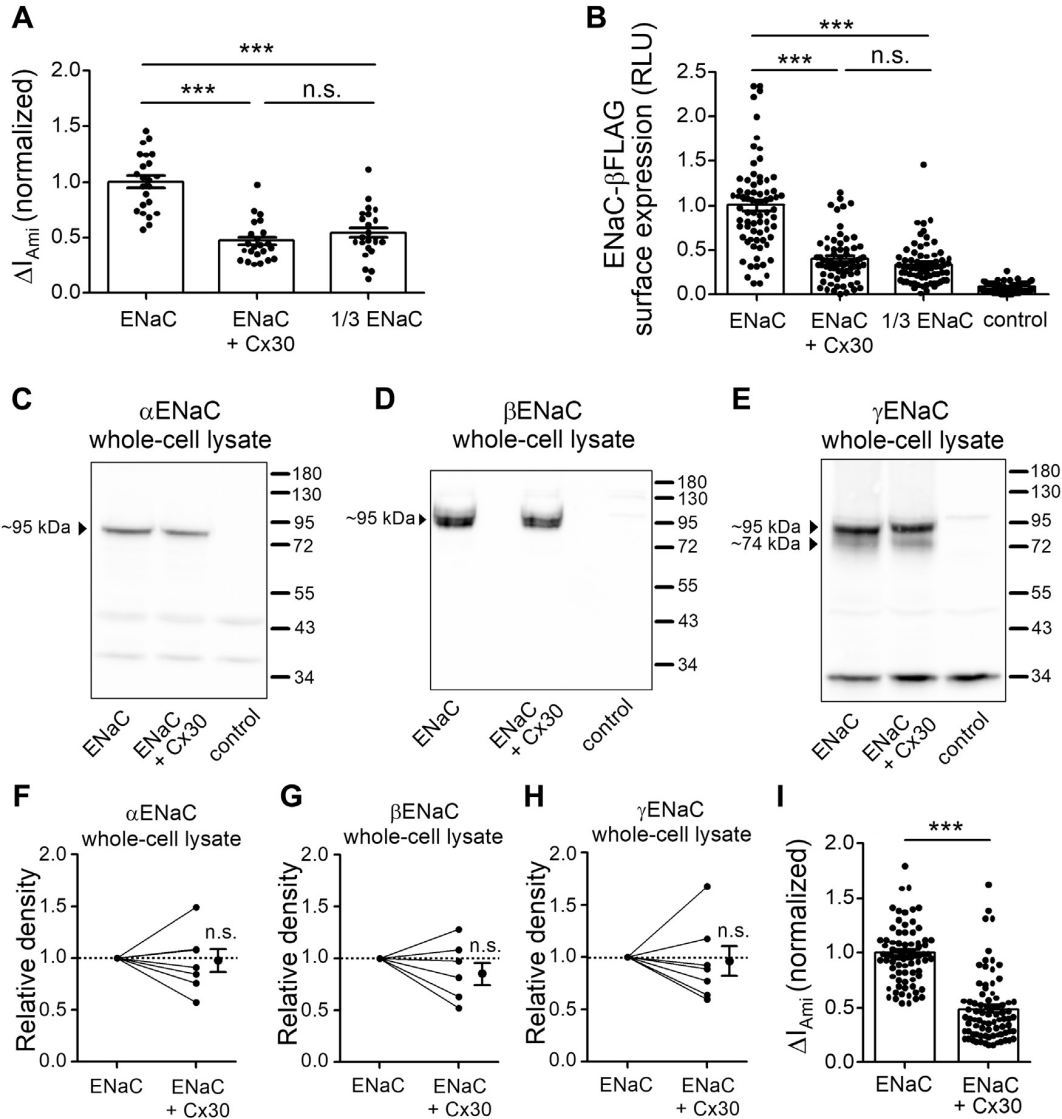
**Inhibition of ENaC currents by Cx30 is due to decreased ENaC expression at the cell surface.***A*, the following groups of oocytes were used: ENaC (injected with 0.6 ng/subunit/oocyte of ENaC cRNA); ENaC+Cx30 (injected with 0.6 ng/subunit/oocyte of ENaC cRNA and 2 ng/oocyte of Cx30 cRNA); 1/3 ENaC (injected with 0.2 ng/subunit/oocyte of ENaC cRNA). To detect channel expression at the cell surface, FLAG-tagged βENaC was coexpressed with wild-type α- and γENaC. ΔI_Ami_ values of individual oocytes were normalized to the mean ΔI_Ami_ measured in oocytes from the corresponding ENaC group. Mean ± SEM and data points for individual oocytes are shown; ∗∗∗*p* < 0.001; n.s. not significant; Kruskall–Wallis with Dunn’s post hoc test (*n* = 23, N = 3). *B*, in parallel with the ΔI_Ami_ measurements shown in A, ENaC-βFLAG surface expression was detected as chemiluminescence signal in relative light units (RLU) using oocytes from the same batch. Control oocytes not expressing ENaC were used to determine the nonspecific background chemiluminescence. Mean ± SEM and data points for individual oocytes are shown; ∗∗∗*p* < 0.001; Kruskall–Wallis with Dunn’s post hoc test (*n* = 70, N = 3). *C*–*E*, representative western blots showing whole-cell expression of αENaC (*C*), βENaC (*D*), or γENaC (*E*) in oocytes expressing ENaC alone (ENaC) or coexpressing ENaC and Cx30. Specific bands for full-length α-, β-, and γENaC at ∼95 kDa (*C*–*E*) and for cleaved γENaC at ∼74 kDa (*E*) were not detected in control oocytes not expressing ENaC. *F*–*H*, densitometric evaluation of full-length bands for αENaC (*F*) and βENaC (*G*) and of full-length and cleaved bands for γENaC (*H*) from similar blots as shown in (*C*–*E*). Density values were normalized in each blot to the signal of αENaC (*F*), βENaC (*G*), or γENaC (*H*) bands obtained from oocytes expressing ENaC alone. *Lines* connect data points obtained in the same experiment, and mean ± SEM are shown; n.s. not significant; one sample Wilcoxon signed-rank test (N = 7). *I*, in parallel experiments to those shown in (*C*–*H*), ΔI_Ami_ was measured to confirm the inhibitory effect of Cx30 on ENaC in these batches of oocytes. Each ΔI_Ami_ value was normalized to the mean ΔI_Ami_ obtained in oocytes expressing ENaC alone. Mean ± SEM and individual data points for each experiment are shown; ∗∗∗*p* < 0.001; Mann–Whitney test (*n* = 81, N = 7).

The inhibitory effect of Cx30 on ENaC may be due to reduced channel expression at the cell surface, reduced single-channel current amplitude, and/or reduced channel open probability. To investigate whether Cx30 changes ENaC surface expression, we used a chemiluminescence assay based on the detection of a FLAG tag inserted into the extracellular domain of the β-subunit of ENaC (62, 63, 64). As shown in Figure 8*A*, coexpression of Cx30 reduced ΔI_Ami_ by about 50% indicating that the inhibitory effect of Cx30 on ENaC including a β-subunit with a FLAG tag (ENaC-βFLAG) was similar to that observed for wild-type ENaC (Fig. 3). Importantly, Cx30 caused a substantial decrease of ENaC-βFLAG surface expression (Fig. 8*B*), which can fully explain the observed ∼50% reduction of ΔI_Ami_. To confirm that the chemiluminescence assay can reliably detect a decrease in channel surface expression under our experimental conditions, we performed control experiments in which the amount of cRNA injected per oocyte was reduced by a factor of 3 (1/3 ENaC). As expected, this significantly reduced both ΔI_Ami_ and the chemiluminescence signal, because fewer channels are expressed and reach the cell surface (Fig. 8, *A* and *B*). The inhibitory effect of reducing the amount of injected cRNA on ΔI_Ami_ and ENaC surface expression was similar to that observed with Cx30 coexpression. In control oocytes, the luminescent signal was very small, which confirmed that the chemiluminescence assay specifically detected ENaC at the cell surface. Additionally, using western blot analysis of whole-cell lysates, we tested whether Cx30 altered total protein expression of α-ENaC (Fig. 8, *C* and *F*), β-ENaC (Fig. 8, *D* and *G*) and γ-ENaC subunits (Fig. 8, *E* and *H*). A single band corresponding to the expected size of full-length α-, β-, and γ-ENaC was detected at about 95 kDa in ENaC-expressing but not in control oocytes. Additionally, a second band corresponding to the partially cleaved γ-ENaC was observed at about 74 kDa. Importantly, densitometric evaluation of ENaC expression demonstrated that the overall protein expression levels of the three ENaC subunits were not significantly altered by Cx30 coexpression (Fig. 8, *F*–*H*). In oocytes from the same batches, the significant inhibitory effect of Cx30 coexpression on ΔI_Ami_ was confirmed (Fig. 8*I*). Thus, Cx30 significantly reduces ENaC expression at the cell surface without detectable effect on overall ENaC protein expression.

### Cx30 stimulates internalization of ENaC

Cx30 may reduce ENaC expression at the cell surface by stimulating ENaC retrieval from the plasma membrane and/or by hampering the insertion of newly synthesized or recycled channels into the plasma membrane. It is well established that mechanisms altering the internalization rate of ENaC have a strong impact on the abundance of ENaC at the cell surface (13, 15, 16, 17, 18, 19, 20, 21). Therefore, we hypothesized that Cx30 decreases ENaC cell surface expression by stimulating ENaC retrieval.

To estimate the rate of ENaC retrieval, we used a strategy similar to that described in previous studies (65, 66). This strategy is based on converting acutely and irreversibly the channels present at the cell surface into fully active channels and on measuring the subsequent current decline as surrogate parameter for the rate of channel retrieval. For this purpose, we used a mutant β-ENaC subunit carrying a cysteine-substitution at the so-called degenerin position (β^S520C^) and coexpressed it with wild-type α- and γ-ENaC with or without additional coexpression of Cx30. This cysteine residue can be covalently modified by application of the sulfhydryl reagent (2-(trimethylammonium)ethyl) methanethiosulfonate bromide (MTSET). This modification by MTSET increases the P_o_ of ENaC at the cell surface close to 1 (67). After MTSET treatment, fully active ENaC channels are continuously retrieved from the cell surface and are gradually replaced by newly inserted unmodified channels with lower P_o_. This results in a decay of ΔI_Ami_ over time toward the initial current level before MTSET application. Thus, measuring the rate of ΔI_Ami_ decline after MTSET treatment allows to estimate the apparent rate of ENaC retrieval from the plasma membrane.

**Figure 9:**
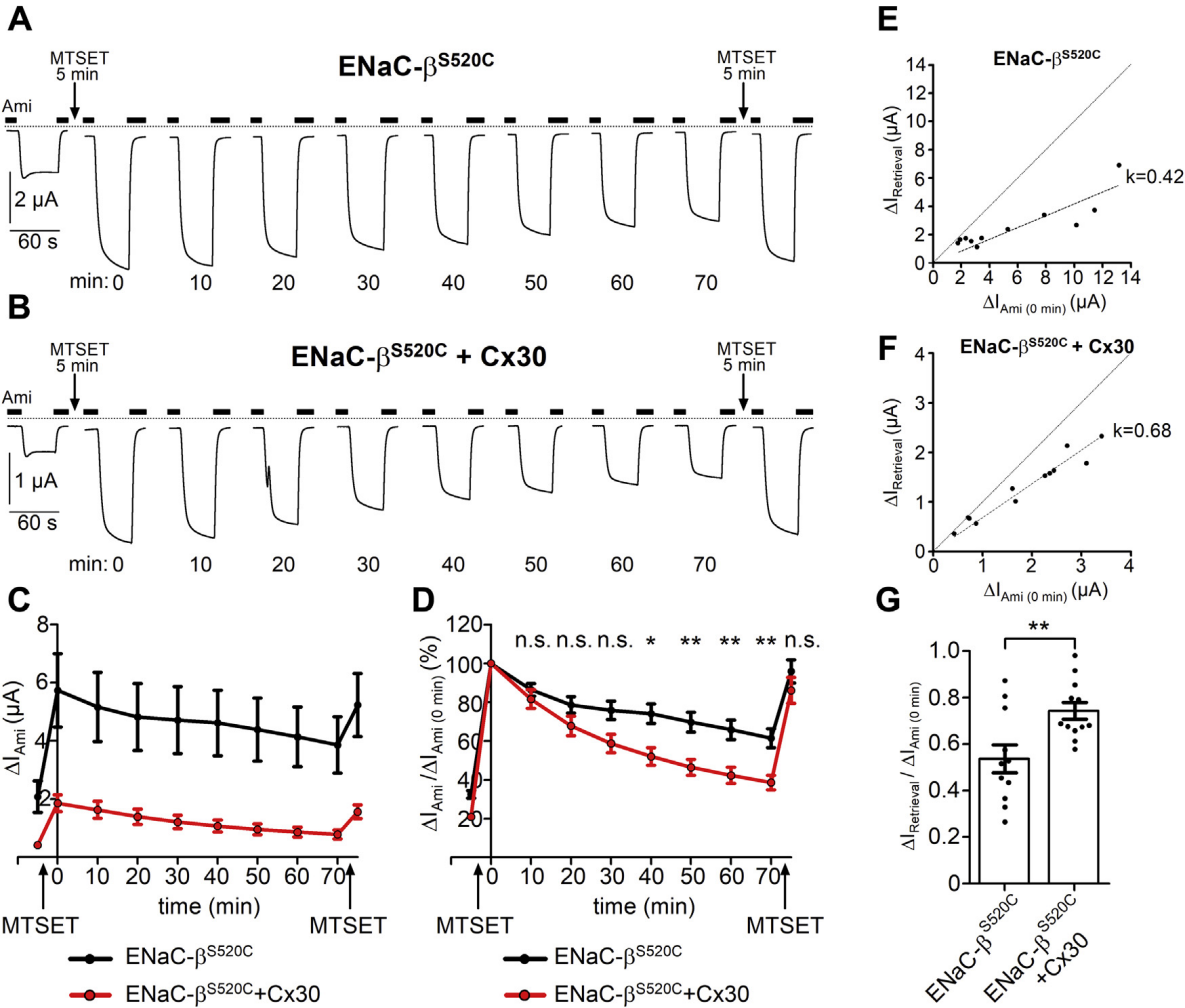
**Cx30 stimulates ENaC internalization.***A* and *B*, representative whole-cell current traces are shown from oocytes expressing wild-type α- and γENaC together with a mutant βENaC subunit carrying a single-point mutation (S520C) without Cx30 (ENaC-β^S520C^, *A*) or with Cx30 coexpression (ENaC-β^S520C^+Cx30, *B*). To determine ΔI_Ami_ at different time points, oocytes were repeatedly clamped for a short period of time at a holding potential of −60 mV and current traces were recorded as illustrated. Oocytes were unclamped during the remaining time of the recording to minimize sodium loading of the oocytes. Impaling microelectrodes were not removed from the oocyte until the end of the experiment. Application of amiloride (Ami, 2 μM) during each current measurement is indicated by *filled bars*. *Dashed lines* indicate zero current level. After determining initial ΔI_Ami_, oocytes were exposed to MTSET for 5 min by exchanging the bath solution from ND96 with 2 μM amiloride to a solution containing in addition 1 mM MTSET (application of MTSET is indicated by *arrows*). Before the second current measurement (time 0 min) MTSET was washed out with ND96 containing 2 μM amiloride. ΔI_Ami_ was determined every 10 min as indicated (min: 10, 20, …, 70). Between the measurements, oocytes were maintained in ND96 with 2 μM amiloride. At the end of the recordings, oocytes were exposed to MTSET for a second time. *C*, summary of ΔI_Ami_ values from similar experiments as shown in the representative traces *A* and *B*. Mean ± SEM current values for ENaC-β^S520C^ oocytes (*black line* and *symbols*; *n* = 11; N = 3) or ENaC-β^S520C^+Cx30 oocytes (*red line* and *symbols*; *n* = 12; N = 3). *D*, data shown in *C* were normalized as ratio of ΔI_Ami_ measured at different time points to ΔI_Ami_ measured at time 0 min after incubation with MTSET (ΔI_Ami_/ΔI_Ami (0 min)_). ∗∗*p* < 0.01; ∗*p* < 0.05; n.s., not significant; ENaC-β^S520C^*versus* ENaC-β^S520C^+Cx30; two-way ANOVA with Bonferroni post hoc test. *E* and *F*, linear regression analysis of ΔI_Retrieval_*versus* ΔI_Ami (0 min)_ in oocytes expressing ENaC alone (*E*) or coexpressing ENaC together with Cx30 (*F*). Each point represents an individual measurement from the same experiments summarized in *C* and *D*. Calculated linear regressions with proportionality coefficients (k) are depicted by *dashed lines*. For better comparison, the diagonal dashed lines depict the hypothetical linear regression with k = 1. *G*, normalized ΔI_Retrieval_ (ΔI_Retrieval_/ΔI_Ami (0 min)_) calculated from the same experiments shown in *E* and *F*. ∗∗*p* < 0.01; Student’s *t*-test.

As illustrated in Figure 9, *A* and *B* and summarized in Figure 9*C*, application of MTSET for 5 min strongly stimulated ΔI_Ami_ in oocytes expressing αβ^S520C^γENaC with or without Cx30 coexpression. In preliminary experiments, we confirmed that application of MTSET for 5 min was sufficient for ΔI_Ami_ to reach a plateau value (*data not shown*). Assuming that MTSET increased ENaC P_o_ close to 1, we estimated a baseline ENaC P_o_ averaging ∼0.2 and ∼0.3 in oocytes with or without coexpression of Cx30, respectively. These values are in good agreement with previously published baseline P_o_ values for human ENaC expressed in oocytes (68, 69, 70) and with the P_o_ values determined in our single channel recordings and estimated from our experiments with chymotrypsin (*see below*). The slightly lower P_o_ value for ENaC in oocytes coexpressing Cx30 may indicate a small inhibitory effect of Cx30 on P_o_. Importantly, the MTSET experiments confirm our conclusion that the large inhibitory effect of Cx30 coexpression on ΔI_Ami_ cannot be attributed to a reduction in P_o_ but must be caused mainly by reduced channel expression at the cell surface. After reaching a maximal value due to exposure to MTSET, ΔI_Ami_ gradually decreased over time in both groups of oocytes. ΔI_Ami_ 70 min after MTSET treatment (ΔI_Ami (70 min)_) in oocytes expressing ENaC alone was reduced to 62 ± 5% of its value measured directly after MTSET treatment (ΔI_Ami (0 min)_). In contrast, in oocytes coexpressing ENaC and Cx30, ΔI_Ami_ decreased within 70 min to 39 ± 4% of its value after MTSET stimulation. Thus, the relative rate of ΔI_Ami_ decline was significantly higher in oocytes coexpressing ENaC and Cx30 compared with that in oocytes expressing ENaC alone (Fig. 9*D*). This finding suggests that Cx30 coexpression stimulates ENaC retrieval from the cell surface.

However, to draw this conclusion, it has to be considered that insertion of newly synthesized and recycled ENaC contributes to ΔI_Ami_ and will partially compensate for its decline due to channel retrieval. Thus, the observed decline of ΔI_Ami_ is likely to underestimate ENaC retrieval rate. Moreover, differences in ENaC insertion rates and P_o_ may at least in part account for the different relative rates of ΔI_Ami_ decline in oocytes with and without Cx30 coexpression. Therefore, to estimate more accurately the contribution of ENaC retrieval to the decline of ΔI_Ami_ within 70 min (ΔI_Retrieval_), it was necessary to correct for the concomitant increase of ΔI_Ami_ resulting from channel insertion (ΔI_Insertion_) during this time period. To make this correction, MTSET was applied again after 70 min assuming that only newly inserted channels can be stimulated by this second MTSET application. As shown in Figure 9, *A*–*D*, in both groups of oocytes, reapplication of MTSET increased ENaC currents almost back to the level reached after the first application of MTSET. This indicates that during the experiment, retrieval of covalently modified channels was balanced by insertion of unmodified channels keeping overall ENaC expression at the cell surface relatively constant. From the current increase caused by the second MTSET application, we estimated ΔI_Insertion_ taking into consideration the increase in P_o_ due to MTSET. ΔI_Retrieval_ was calculated using the following equation (see Experimental procedures):

$$\Delta {\text{I}}_{\text{Retrieval}}\;=\;\Delta {\text{I}}_{\text{Ami}\left(\text{0}\;\text{min}\right)}-\left(\Delta {\text{I}}_{\text{Ami}\left(\text{70}\;\text{min}\right)}-\Delta {\text{I}}_{\text{Insertion}}\right)$$

This correction revealed that without concomitant channel insertion, ΔI_Ami_ would have been reduced by ENaC retrieval within 70 min to 46 ± 6% (*n* = 11; N = 3) of ΔI_Ami (0 min)_ in oocytes expressing ENaC alone and to 26 ± 4% (*n* = 12; N = 3; *p* < 0.01) in oocytes coexpressing ENaC and Cx30. Using these corrected values, we performed a regression analysis and demonstrated that ΔI_Retreival_ correlated linearly with ΔI_Ami (0 min)_ in both groups of oocytes (Fig. 9, *E* and *F*), which indicates that their endocytic machinery was not saturated. Importantly, in oocytes coexpressing ENaC and Cx30, the linear regression coefficient was higher (k = 0.68 ± 0.02; Fig. 9*F*) than that in oocytes expressing ENaC alone (k = 0.42 ± 0.04; Fig. 9*E*). Accordingly, the normalized ΔI_Retrieval_ was significantly higher with Cx30 than without Cx30 (Fig. 9*G*).

In summary, this further supports the conclusion that Cx30 coexpression significantly stimulates the rate of ENaC retrieval from the plasma membrane, thereby decreasing channel expression at the cell surface.

### Cx30 has no effect on proteolytic activation of ENaC

**Figure 10:**
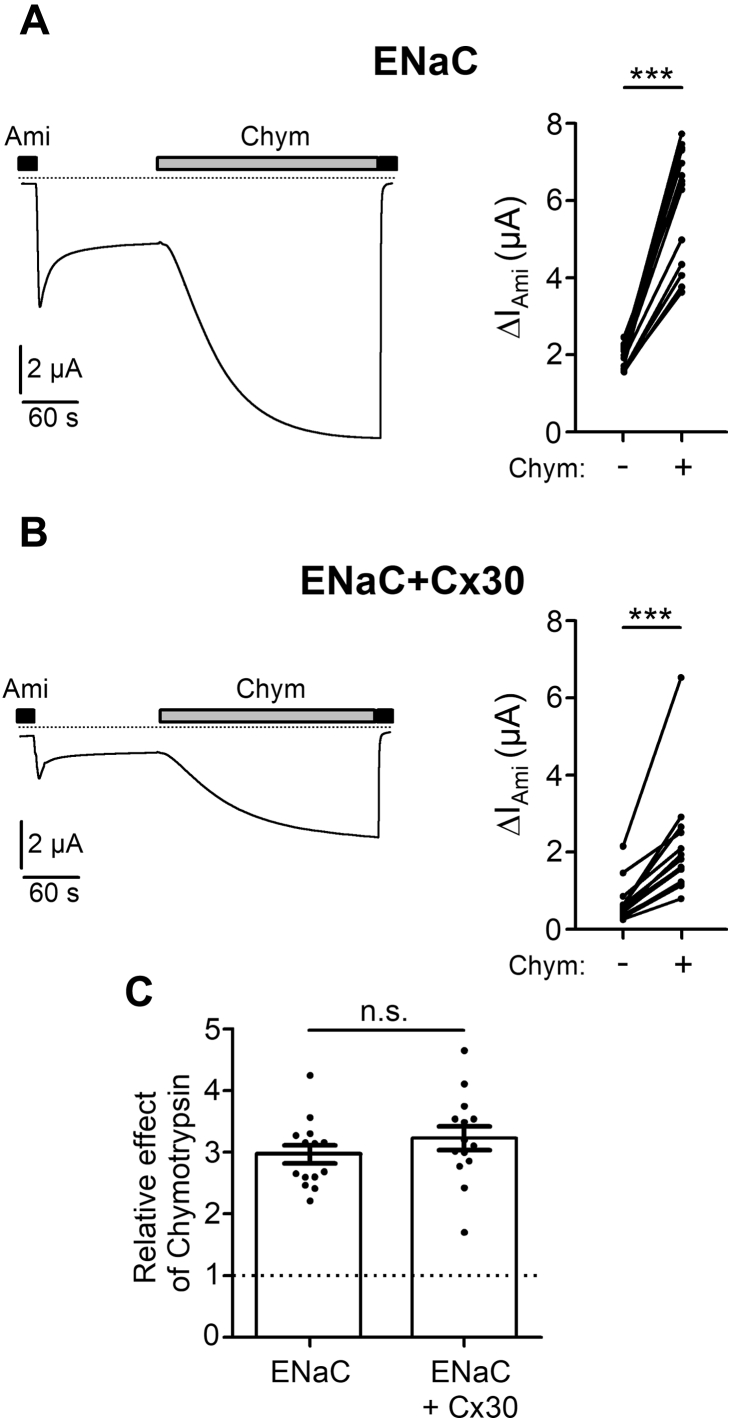
**In oocytes coexpressing ENaC and Cx30, the stimulatory effect of chymotrypsin on ENaC currents is similar to that in oocytes expressing ENaC alone**. *A* and *B*, *Left panels*, representative whole-cell current traces recorded in an oocyte expressing ENaC alone (*A*) and in an oocyte coexpressing ENaC and Cx30 (*B*). Application of amiloride (Ami, 2 μM) or chymotrypsin (2 μg/ml) is indicated by *filled* and *gray bars*, respectively. *Dashed lines* indicate zero current level. *Right panels*, ΔI_Ami_ values obtained from similar experiments as shown in the representative traces (*left panels*) before (−) and after (+) chymotrypsin application. *Lines* connect data points obtained in an individual oocyte; ∗∗∗*p* < 0.001; Wilcoxon matched-pairs signed-rank test (*n* = 14, N = 2). *C*, summary of the individual data shown in (*A* and *B*) normalized as relative stimulatory effect of chymotrypsin on ΔI_Ami_. Mean ± SEM and data points for individual oocytes are shown; n.s., not significant; Student’s ratio *t*-test.

To investigate a possible effect of Cx30 on proteolytic ENaC activation, we used chymotrypsin (2 μg/ml) to activate ENaC in oocytes expressing ENaC with or without Cx30. We have previously shown that chymotrypsin in this concentration maximally activates ENaC by proteolytic cleavage, increasing average channel open probability (P_o_) from about 0.3 to almost 1 consistent with a threefold increase of ENaC-mediated whole-cell currents (64, 68, 71). As expected, washout of amiloride revealed larger ENaC-mediated baseline currents in oocytes expressing ENaC alone (Fig. 10*A*) than in oocytes coexpressing Cx30 and ENaC (Fig. 10*B*) confirming the inhibitory effect of Cx30 on ENaC. Importantly, chymotrypsin stimulated baseline ENaC currents in both groups of oocytes by approximately, threefold (Fig. 10*C*). This indicates that Cx30 does not interfere with proteolytic ENaC activation and probably has no major effect on channel P_o_, which was about 0.3 in both groups.

### Coexpression of Cx30 does not affect the single-channel current amplitude of ENaC and has no apparent effect on single-channel P_o_

**Figure 11:**
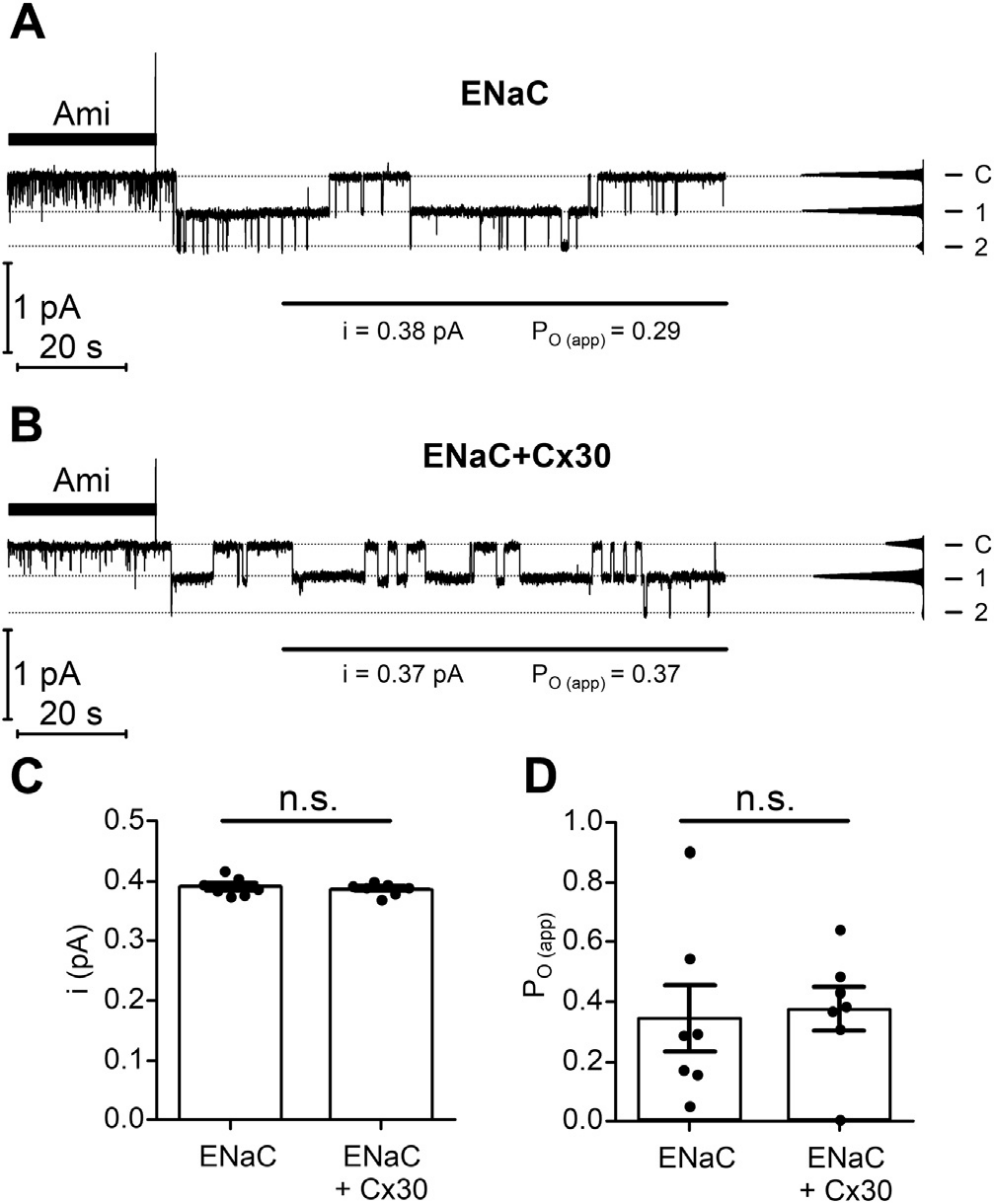
**Coexpression of Cx30 has no apparent effect on the single-channel current amplitude and open probability of ENaC.***A* and *B*, representative continuous single-channel current recordings in an outside-out patch of an ENaC expressing oocyte (*A*) or an ENaC+Cx30 coexpressing oocyte (*B*) obtained at a holding potential of −70 mV. Application of amiloride (2 μM) is indicated by *filled bars*. The current level at which all channels are closed (C) was determined in the presence of amiloride. The channel open levels are indicated by (1) and (2). Single-channel binned current amplitude histograms were obtained by analyzing an 85 s portion of the current trace indicated by a horizontal line and were used to calculate single-channel current amplitude (i) and P_O (app)_ values. *C* and *D*, summary of data from similar experiments as shown in *A* and *B*. Mean ± SEM and data points for individual oocytes are shown; n.s., not significant; Mann–Whitney test (*n* = 7, N = 4).

To test whether Cx30 expression alters the single-channel current amplitude of ENaC, we performed single-channel recordings in outside-out patches of oocytes expressing ENaC alone or ENaC together with Cx30. Figure 11, *A* and *B* show representative recordings from an outside-out patch excised from an oocyte expressing only ENaC or ENaC with Cx30, respectively. The initial washout of amiloride revealed single-channel activity with up to two apparent channel levels. The single-channel current amplitude was about 0.38 pA, which is typical for ENaC at this holding potential (and corresponds to a single-channel conductance of about 5 pS). Importantly, the average single-channel current amplitude was not significantly different in ENaC *versus* ENaC+Cx30 expressing oocytes (Fig. 11*C*). The estimated apparent single-channel P_o_ in these recordings was rather variable, which is a typical finding in single-channel recordings of ENaC. On average, ENaC P_o_ was similar in both groups (Fig. 11*D*). However, due to the variability of single-channel P_o_, we cannot exclude the possibility that Cx30 has a minor inhibitory effect on ENaC P_o_ as suggested by our experiments with MTSET (see above; Fig. 9). Collectively, our data indicate that the inhibitory effect of Cx30 is mainly mediated by a reduction of the number of ENaC channels in the plasma membrane and not by an effect on channel P_o_ or single-channel conductance.

### C-termini of β- and γ-ENaC are critically involved in Cx30-mediated downregulation of ENaC

**Figure 12:**
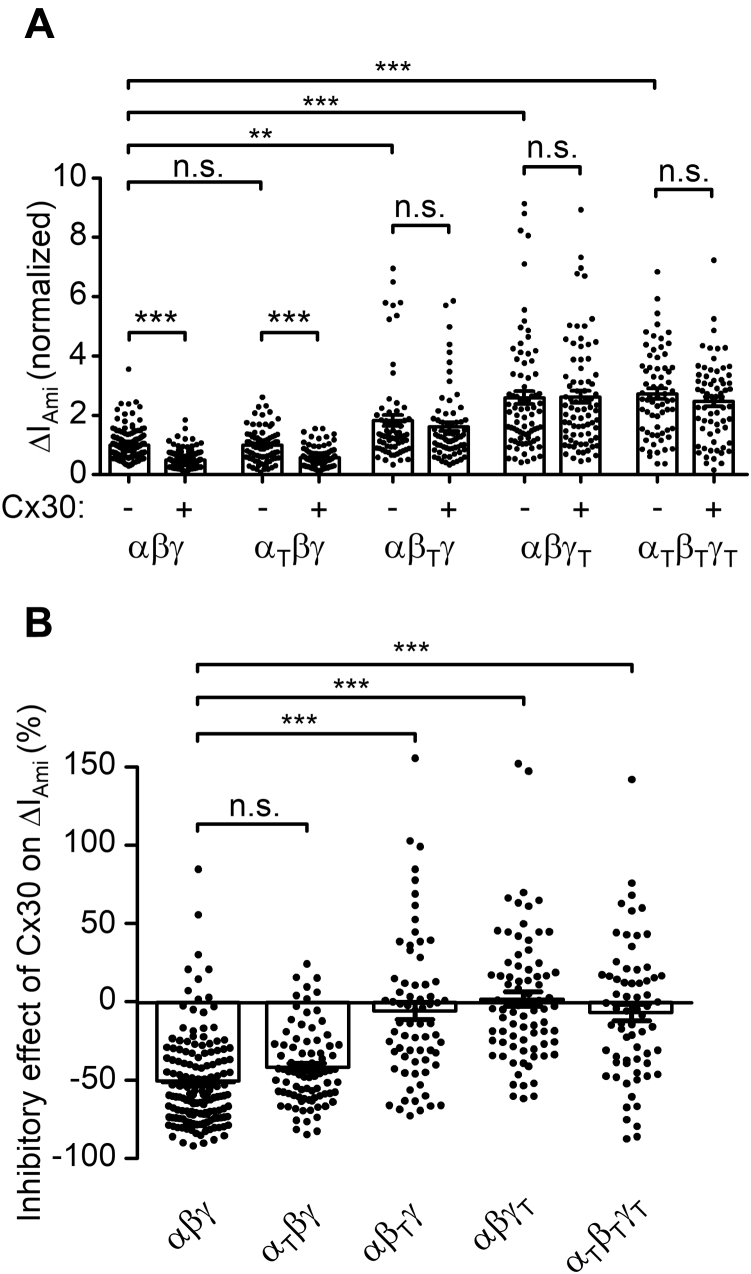
**Intact C-termini of β- and γENaC are necessary for the inhibitory effect of Cx30 on ENaC.***A*, normalized ΔI_Ami_ values are shown from oocytes expressing wild-type ENaC (αβγ), truncated αENaC (P619X), βENaC (R566X), or γENaC (K576X) with corresponding wild-type ENaC subunits (α_T_βγ, αβ_T_γ or αβγ_T_) or triple-truncated ENaC (α_T_β_T_γ_T_) in each case with or without Cx30. Individual ΔI_Ami_ values were normalized to the mean ΔI_Ami_ obtained in matched oocytes from the same batch expressing wild-type ENaC without Cx30. Mean ± SEM and data points for individual oocytes are shown; ∗∗∗*p* < 0.001; ∗∗*p* < 0.01; n.s. not significant; Kruskall–Wallis with Dunn’s post hoc test (67 ≤ *n* ≤ 153, 5 ≤ N ≤ 7). *B*, relative inhibitory effect of Cx30 on wild-type (αβγ) or truncated (α_T_βγ, αβ_T_γ, αβγ_T_ or α_T_β_T_γ_T_) ENaC calculated as described in Figure 3 using original data shown in *A*. ΔI_Ami_ obtained in oocytes expressing wild-type (αβγ) or truncated (α_T_βγ, αβ_T_γ, αβγ_T_, or α_T_β_T_γ_T_) ENaC together with Cx30 was normalized to the corresponding mean ΔI_Ami_ recorded in matched control oocytes from the same batch expressing wild-type (αβγ) or truncated (α_T_βγ, αβ_T_γ, αβγ_T_ or α_T_β_T_γ_T_) ENaC alone. Mean ± SEM and data points for individual oocytes are shown; ∗∗∗*p* < 0.001; n.s., not significant; Kruskall–Wallis with Dunn’s post hoc test.

It is well documented that C-termini of ENaC subunits (β- and γENaC in particular) are critically involved in the regulation of channel retrieval from the plasma membrane (4). Therefore, we tested whether the C-termini of α-, β-, or γ-ENaC are involved in the Cx30-mediated downregulation of ENaC. For this purpose, channels with one C-terminally truncated ENaC subunit (α_T_: P619X, β_T_: R566X, or γ_T_: K576X) and two wild-type subunits (α_T_βγ, αβ_T_γ, or αβγ_T_) were expressed alone or coexpressed with Cx30. The truncations eliminated highly conserved C-terminal sequences (72). As shown in Figure 12*A*, C-terminal truncations of β- or γENaC significantly increased ENaC currents compared with the wild-type channel. Truncation of all three C-termini had a similar stimulatory effect as that of truncating the C-terminus of the γ-subunit alone. These findings are consistent with the well-established concept that channels with C-terminal truncations are internalized and degraded more slowly than wild-type ENaC. Interestingly, ΔI_Ami_ was not increased in oocytes expressing α_T_ENaC, which is in agreement with previously reported findings that C-terminal truncations of β- or γENaC but not of αENaC substantially increased ENaC-mediated currents (15, 72). The relative inhibitory effect of Cx30 on ENaC-mediated currents was preserved in oocytes expressing α_T_βγENaC (42 ± 3%) and was similar to that observed in matched oocytes expressing wild-type ENaC (52 ± 2%; Fig. 12*B*). In contrast, the relative inhibitory effect of Cx30 on ΔI_Ami_ was significantly reduced in oocytes expressing αβ_T_γENaC (17 ± 5%) and essentially abolished in oocytes expressing αβγ_T_ENaC (0 ± 5%) or α_T_β_T_γ_T_ENaC (4 ± 4%; Fig. 12*B*). In additional control experiments, it was confirmed that in oocytes coexpressing Cx30 and αβγ_T_ENaC, the Cx30-mediated inward currents elicited by Ca^2+^ and Mg^2+^ removal were similar to those observed in matched oocytes coexpressing Cx30 and wild-type ENaC (*data not shown*). Thus, the absence of the inhibitory effect of Cx30 on αβγ_T_ENaC was not due to impaired expression of Cx30 hemichannels at the cell surface. In conclusion, these data indicate that Cx30-mediated downregulation of ENaC critically depends on the presence of intact C-termini of β- and γ-ENaC.

### Nedd4-2 is not involved in Cx30-mediated ENaC downregulation

**Figure 13:**
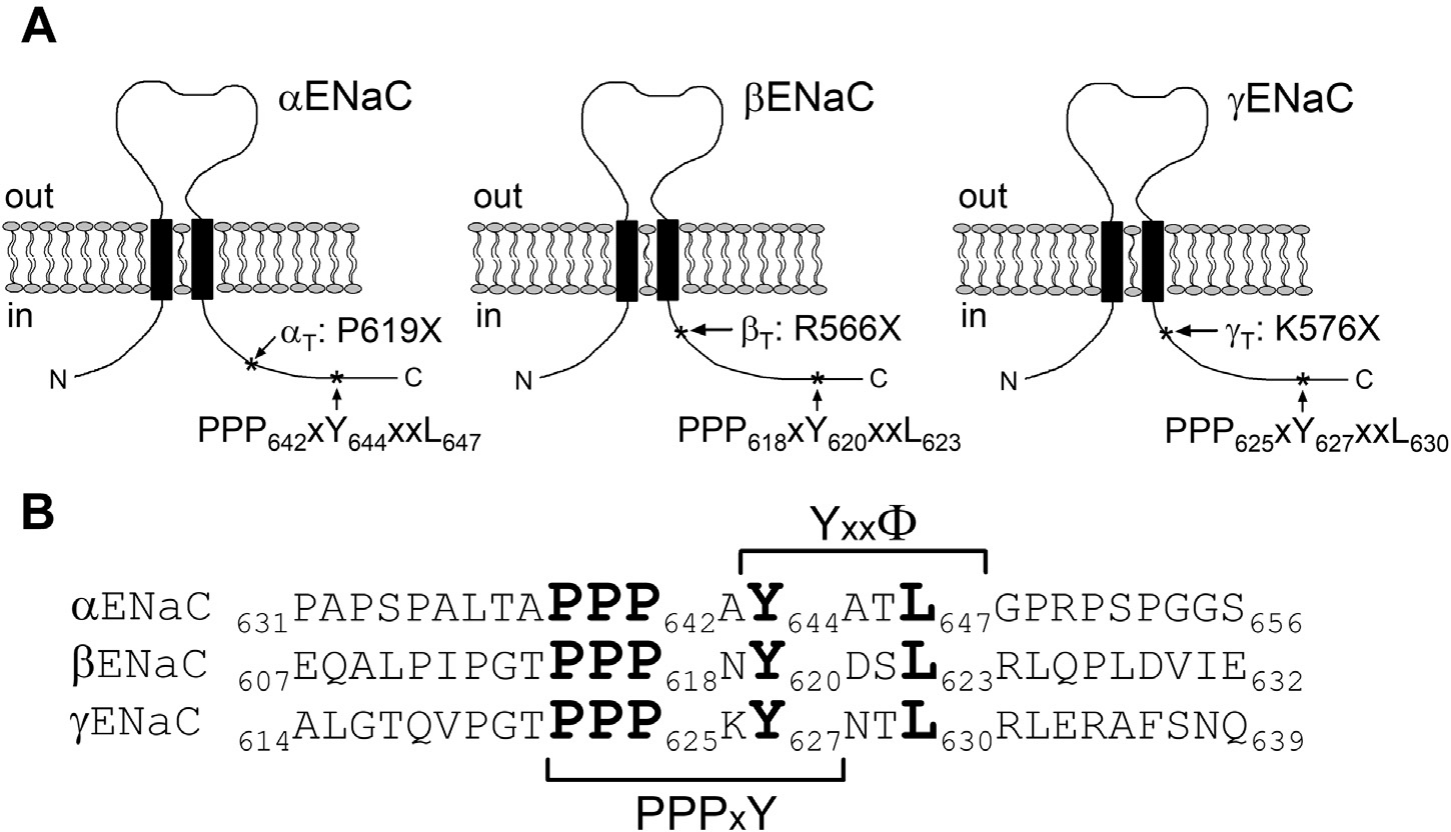
**C-termini of ENaC subunits have overlapping PPPxY- and YxxΦ-motifs.***A*, schematic representation of α, β, and γENaC illustrating the extracellular loop, two transmembrane domains (*black rectangles*), and intracellular N- and C-termini. The position within the C-terminus of each subunit at which a truncation mutation was introduced and the localization of the highly conserved PPPxYxxL sequence are indicated by *asterisks*. *B*, primary sequence alignment of the corresponding C-terminal regions of human αβγENaC. Highly conserved amino acid residues belonging to PPPxY- and YxxΦ-motifs are highlighted in **bold**.

**Figure 14:**
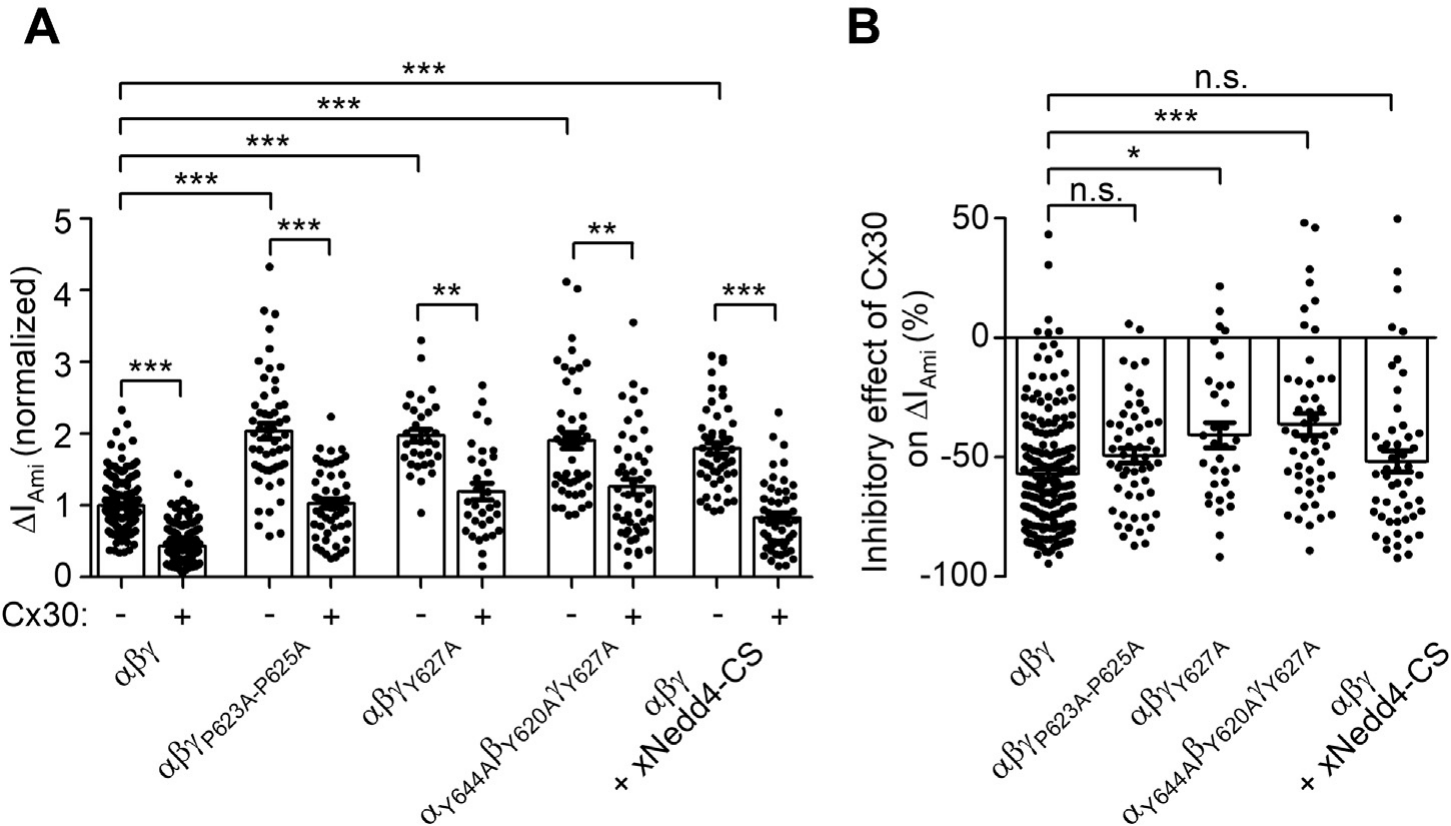
**ENaC inhibition by Cx30 does not involve a Nedd4-2-dependent mechanism.***A*, ΔI_Ami_ values obtained in oocytes expressing wild-type ENaC (αβγ), mutant ENaC (αβγ_P623A-P625A_, αβγ_Y627A_, α_Y644A_β_Y620A_γ_Y627A_), or coexpressing wild-type ENaC together with xNedd4-CS (αβγ + xNedd4-CS) in each case with (+) or without (−) additional coexpression of Cx30 were normalized as described in Figure 12*A*. Mean ± SEM and data points for individual oocytes are shown; ∗∗∗*p* < 0.001; ∗∗*p* < 0.01; Kruskall–Wallis with Dunn’s post hoc test (32 ≤ *n* ≤ 185, 3 ≤ N ≤ 4). *B*, relative inhibitory effect of Cx30 on wild-type ENaC (αβγ), mutant ENaC (αβγ_P623A-P625A_, αβγ_Y627A_, α_Y644A_β_Y620A_γ_Y627A_), or wild-type ENaC coexpressed with xNedd4-CS (αβγ + xNedd4-CS) calculated as described in Figure 12*B* using original data shown in *A*. Mean ± SEM and data points for individual oocytes are shown; ∗*p* < 0.05; ∗∗∗*p* < 0.001; n.s., not significant; Kruskall–Wallis with Dunn’s post hoc test.

Sequence alignment of ENaC C-termini (Fig. 13) reveals the presence of highly conserved PPPxY-motifs in all three ENaC subunits. The role of these PPPxY-motifs in the regulation of ENaC cell surface expression has been characterized in detail (13, 15, 16, 19, 20, 21). The PPPxY-motifs are recognized by WW domains of the ubiquitin ligase Nedd4-2, which leads to Nedd4-2 binding to ENaC and subsequent channel ubiquitination and internalization. Importantly, mutating the PPPxY-motifs in β- or γ-ENaC increased ENaC-mediated currents to a similar degree as truncating the corresponding C-termini (15, 16). Therefore, we investigated whether mutating the PPPxY-motif in γENaC also abolished the inhibitory effect of Cx30 on ENaC. In oocytes expressing a mutant channel with the three critical proline residues (_623_PPP_625_) in the PPPxY-motif of γENaC mutated to alanine (αβγ_P623A-P625A_), average ΔI_Ami_ was almost doubled compared with ΔI_Ami_ in matched oocytes expressing wild-type ENaC (Fig. 14*A*). This confirmed previously reported data and is consistent with the concept that mutating the PPPxY-motif of γENaC impedes the interaction of endogenous Nedd4 with ENaC. However, the relative inhibitory effect of Cx30 on ΔI_Ami_ was not significantly different in oocytes expressing γ_P623A-P625A_ mutant ENaC (49 ± 3%) compared with that observed in oocytes expressing wild-type ENaC (57 ± 2%) (Fig. 14*B*). This finding suggests that Nedd4-2 is not involved in Cx30-mediated ENaC downregulation. In further experiments, we mutated the tyrosine residue (Y627) in the PPPxY-motif of γENaC to alanine, which is also thought to be critical for Nedd4-2 binding to ENaC (13, 19, 20, 21). In good agreement with this concept, we found that the γ_Y627A_ mutation increased ΔI_Ami_ to a similar extent as the γ_P623A-P625A_ mutation (Fig. 14*A*). Interestingly, the relative inhibitory effect of Cx30 on γ_Y627A_ mutant ENaC was slightly reduced (41 ± 5%) compared with that on wild-type ENaC (Fig. 14*B*). Mutating the homologous tyrosine residues in all three ENaC subunits (α_Y644A_β_Y620A_γ_Y627A_ENaC) resulted in even more pronounced reduction of Cx30-mediated effect on ENaC (36 ± 5%). Thus, disruption of the PPPxY-motif by alanine substitutions of the proline residues in γENaC did not significantly alter the inhibitory effect of Cx30 on ENaC. In contrast, substitution of the tyrosine residue by an alanine had a small but significant effect on Cx30-mediated ENaC inhibition. We also investigated the effect of coexpressing catalytically inactive *Xenopus* xNedd4-CS (bearing a C938S mutation in the Hect domain) on Cx30-mediated ENaC downregulation. As expected from previously reported findings (73), coexpression of xNedd4-CS significantly increased ENaC-mediated currents by about approximately twofold probably due to an inhibitory effect on endogenous Nedd4 (Fig. 14*A*). However, the relative inhibitory effect of Cx30 on ΔI_Ami_ was not significantly affected by coexpressing xNedd4-CS (52 ± 4%; Fig. 14*B*). Taken together, these data support the conclusion that Nedd4-2 is not involved in mediating the inhibitory effect of Cx30 on ENaC.

### Cx30-mediated ENaC downregulation is significantly reduced by inhibiting clathrin-mediated endocytosis or mutating the YxxΦ-motif in the C-termini of β- or γ-ENaC

Primary sequence alignment of ENaC C-termini (Fig. 13) reveals that all three ENaC subunits have highly conserved YxxΦ-motifs (where Φ indicates an amino acid with a bulky hydrophobic side chain like leucine), which partially overlap with their PPPxY-motifs. YxxΦ-motifs are known to be involved in binding the μ-subunit of the clathrin adaptor protein 2 (AP-2), which is responsible for clathrin-mediated endocytosis of many cell surface proteins (26, 28, 29). Thus, the YxxΦ-motifs are likely to be important for clathrin-mediated ENaC internalization and may be involved in mediating the inhibitory effect of Cx30 on ENaC.

**Figure 15:**
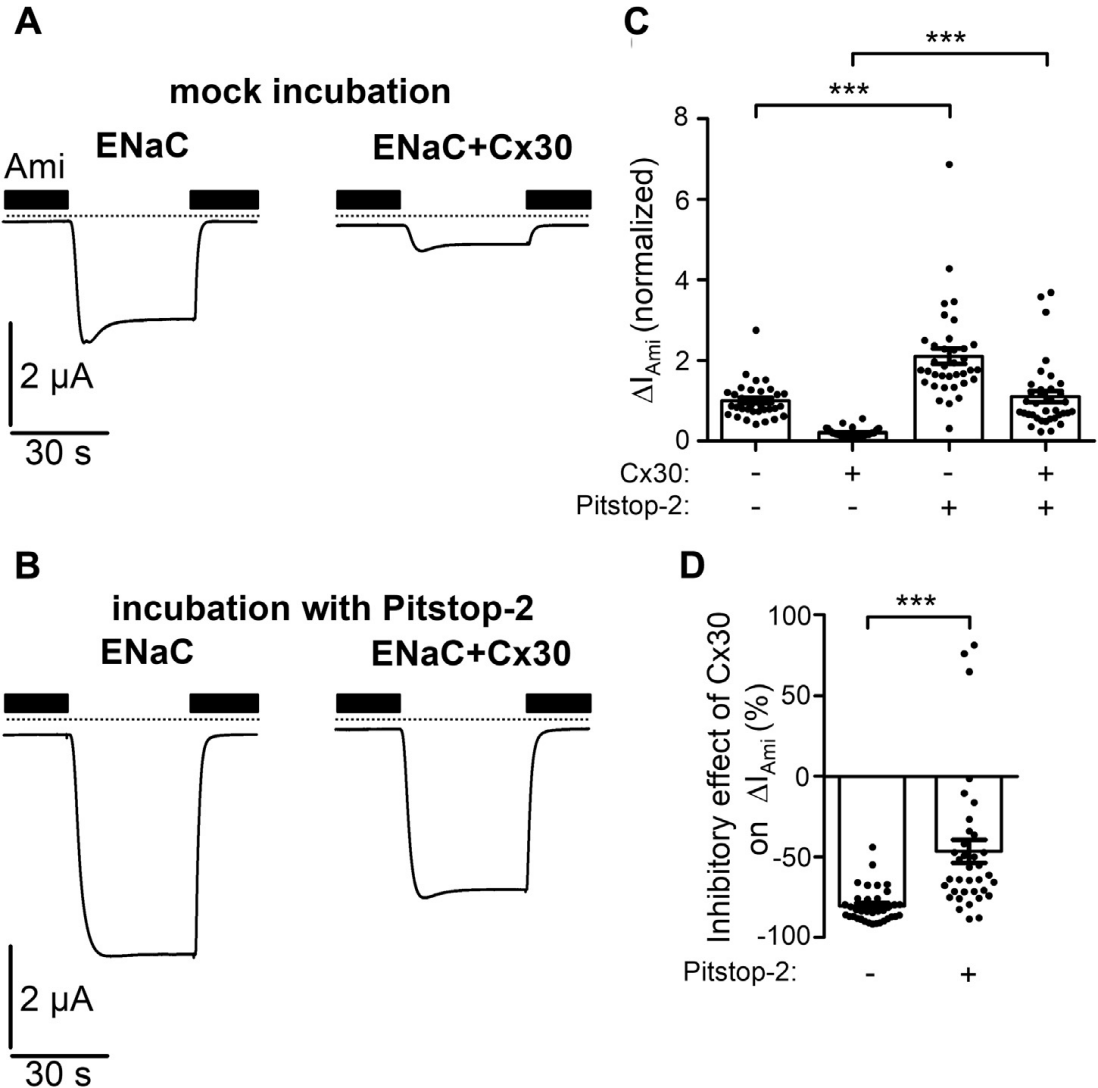
**Inhibition of clathrin-mediated endocytosis significantly decreases the inhibitory effect of Cx30 on ENaC.***A* and *B*, representative whole-cell current traces recorded in oocytes expressing ENaC alone (*left panels*) or coexpressing ENaC with Cx30 (*right panels*) incubated for 1 h in standard incubation solution containing 0.02% DMSO (*A*; mock incubation) or 30 μM Pitstop-2 with 0.02% DMSO (*B*; Pitstop-2 was dissolved in DMSO to prepare a stock solution). Application of amiloride (Ami, 2 μM) is indicated by *filled bars*. *Dashed lines* indicate zero current level. *C*, ΔI_Ami_ values from similar experiments as shown in the representative traces in *A* and *B*. Mean ± SEM and data points for individual oocytes are shown; ∗∗∗*p* < 0.001; Kruskall–Wallis with Dunn’s post hoc test (*n* = 36 for each group, N = 3). *D*, relative inhibitory effect of Cx30 on ENaC calculated from the data shown in *C* essentially as described in Figure 3. DMSO, dimethyl sulfoxide. Mean ± SEM and data points for individual oocytes are shown; ∗∗∗*p* < 0.001; Mann–Whitney test.

**Figure 16:**
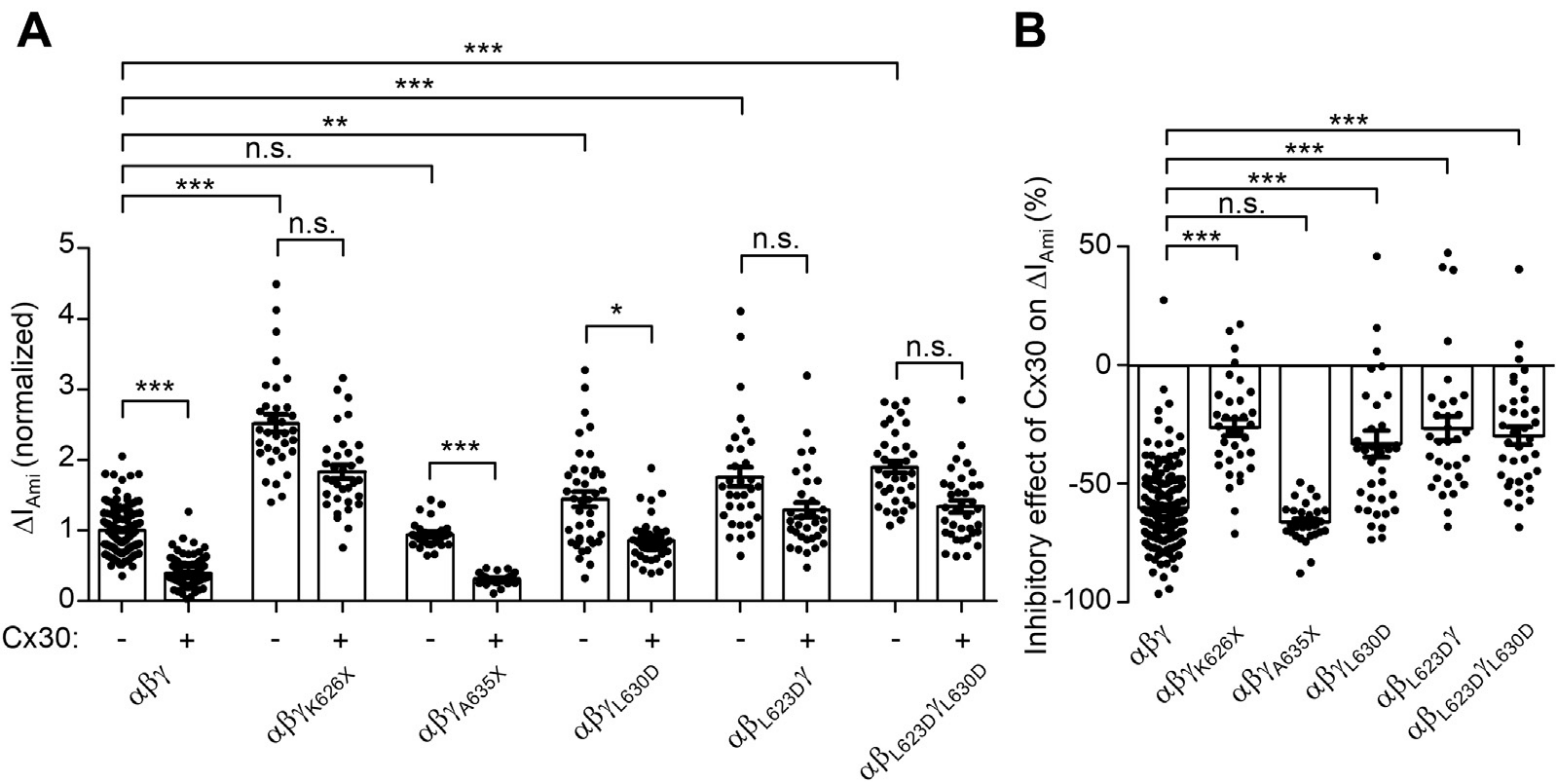
**YxxΦ-motif of β- and γENaC is critically involved in Cx30-mediated ENaC inhibition.***A*, ΔI_Ami_ values obtained in oocytes expressing wild-type (αβγ), truncated (αβγ_K626X_, αβγ_A635X_), or mutant (αβγ_L630D_, αβ_L623D_γ, αβ_L623D_γ_L630D_) ENaC with or without Cx30 were normalized as described in Figure 12*A*. Mean ± SEM and data points for individual oocytes are shown; ∗∗∗*p* < 0.001; ∗∗*p* < 0.01; n.s., not significant; Kruskall–Wallis with Dunn’s post hoc test (30 ≤ *n* ≤ 172, 3 ≤ N ≤ 4). *B*, relative inhibitory effect of Cx30 on wild-type (αβγ), truncated (αβγ_K626X_, αβγ_A635X_), or mutant (αβγ_L630D_, αβ_L623D_γ, αβ_L623D_γ_L630D_) ENaC calculated as described in Figure 12*B* using original data shown in (*A*). Mean ± SEM and data points for individual oocytes are shown; ∗∗∗*p* < 0.001; n.s., not significant; Kruskall–Wallis with Dunn’s post hoc test.

To explore a possible role of clathrin-mediated endocytosis in Cx30-mediated inhibition of ENaC, we used a pharmacological tool, Pitstop-2, which specifically binds to clathrin, thereby inhibiting clathrin-mediated endocytosis (74). Oocytes expressing ENaC with or without Cx30 were preincubated with Pitstop-2 (30 μM) or with 0.02% dimethyl sulfoxide (mock incubation) for 1 h before determining ΔI_Ami_. In ENaC expressing oocytes without coexpression of Cx30, Pitstop-2 treatment significantly increased ΔI_Ami_ by about twofold compared with ΔI_Ami_ measured in oocytes after mock incubation (Fig. 15, *A* and *C*). This increase of ΔI_Ami_ may be explained by an effective inhibition of clathrin-mediated ENaC retrieval by Pitstop-2 while channel insertion into the plasma membrane continues to occur resulting in a net increase of channel expression at the cell surface. Importantly, in oocytes coexpressing ENaC with Cx30, the relative stimulatory effect of Pitstop-2 on ΔI_Ami_ was much more pronounced with an average increase of about fivefold (Fig. 15, *B* and *C*). Moreover, ΔI_Ami_ values in Pitstop-2-treated oocytes coexpressing ENaC and Cx30 reached a level similar to that observed in mock-treated oocytes expressing ENaC alone. Thus, inhibition of clathrin-mediated endocytosis by Pitstop-2 significantly reduced the inhibitory effect of Cx30 on ENaC (Fig. 15*D*). Taken together these findings indicate that clathrin-mediated endocytosis is critically involved in the downregulation of ENaC by Cx30. Next, we introduced mutations in γENaC resulting in truncations before (γ_K626X_) or after (γ_A635X_) the YxxΦ-motif. Importantly, deleting the YxxΦ-motif by the γ_K626X_ mutation increased ΔI_Ami_ and significantly reduced the relative inhibitory effect of Cx30 on ENaC (Fig. 16). In contrast, truncating the C terminus after the YxxΦ-motif (γ_A635X_) affected neither the basal ΔI_Ami_ nor the relative inhibitory effect of Cx30 on ENaC (Fig. 16). These data indicate that the intact YxxΦ-motif in γENaC is critically important for the Cx30-mediated ENaC inhibition. To investigate this further, we substituted the critical leucine (γ_L630_) to a negatively charged aspartate (γ_L630D_). This increased basal ΔI_Ami_ (by about 44%, Fig. 16*A*) and reduced the degree of Cx30-mediated ENaC inhibition (Fig. 16*B*). Substitution of the homologous leucine in the YxxΦ-motif of β-ENaC to aspartate (β_L623D_) or the simultaneous substitution of both leucine residues in β- and γ-ENaC (β_L623D_γ_L630D_) also increased basal ΔI_Ami_ and reduced the inhibitory effect of Cx30 on ENaC (Fig. 16). Thus, our data highlight the crucial role of the C-terminal YxxΦ-motifs of β- and γENaC in Cx30-mediated ENaC inhibition. Collectively, our findings demonstrate that enhanced clathrin-mediated channel retrieval is mainly responsible for the inhibitory effect of Cx30 on ENaC.

## Discussion

In the current paper, we investigated the functional interaction of human αβγENaC with human Cx30 heterologously expressed in *X. laevis* oocytes. We made the following observations: 1) coexpression of Cx30 significantly reduced ENaC-mediated currents by decreasing channel surface expression due to enhanced channel retrieval; 2) Cx30 had no noticeable effect on proteolytic activation and single-channel properties of ENaC; 3) ENaC inhibition by Cx30 was prevented by inhibiting Cx30 channel function with carbenoxolone or a point mutation; 4) the inhibitory effect of Cx30 depended on the C-termini of β- and γENaC without involving a Nedd4-2-dependent mechanism; 5) inhibition of clathrin-mediated endocytosis with Pitstop-2 or by mutating putative AP-2 recognition motifs (YxxФ) in the C-termini of β- and/or γENaC significantly reduced the inhibitory effect of Cx30 on ENaC. These findings indicate that enhanced clathrin-mediated channel retrieval from the cell surface is the major mechanism of ENaC inhibition by Cx30.

Previously, it has been proposed that Cx30 hemichannels modulate ENaC activity in the mouse distal tubule by mediating flow-sensitive release of ATP mainly from intercalated cells (34, 47, 75). The released ATP is thought to inhibit ENaC in principal cells in an autocrine/paracrine manner *via* P2Y_2_-dependent purinergic signaling causing a rise in intracellular Ca^2+^. However, endogenous expression of purinergic receptors, and hence the responsiveness to extracellular ATP, is negligible in *X. laevis* oocytes (76, 77). Moreover, it has been reported that application of ATP does not inhibit ENaC-mediated currents in the oocyte expression system (78). This argues against a role of Cx30-mediated ATP release in mediating the inhibitory effect of Cx30 on ENaC observed in the present study, but does not rule out the possibility that purinergic signaling contributes to the inhibitory effect of Cx30 on ENaC in native renal tubules. Interestingly, Cx30 hemichannels mediate not only the efflux of ATP but also of a variety of other signaling molecules including NAD^+^, glutathione, glutamate, PGE2, or polyamines (43, 44, 45). Thus, it is conceivable that in addition to ATP, other signaling molecules released by Cx30 may affect ENaC activity. Future studies are needed to explore the potential relevance of such factors.

Expression of Cx30 hemichannels in oocytes resulted in the appearance of a small carbenoxolone-sensitive cation conductance in the presence of divalent cations. This indicates that Cx30 has residual hemichannel activity in the plasma membrane even in the presence of physiological concentrations of Ca^2+^ and Mg^2+^ in the extracellular solution. Inhibiting Cx30 channel activity by carbenoxolone largely prevented the inhibitory effect of Cx30 on ENaC. Moreover, a point mutation (M34A) that abolishes Cx30 ion channel function, without affecting its intracellular and cell surface expression, prevented its inhibitory effect on ENaC. These findings indicate that channel activity of Cx30 is required for its inhibitory effect on ENaC. Moreover, increasing the Ca^2+^ concentration from 1.8 mM to 3.6 mM in the incubation medium significantly reduced the inhibitory effect of Cx30 coexpression on ENaC, whereas decreasing the Ca^2+^ concentration from 1.8 mM to 0.9 mM had an opposite effect. This further supports the conclusion that the inhibitory effect of Cx30 depends on its ion channel function, because it is known to be inhibited by extracellular Ca^2+^ (61). The reduction of Cx30 hemichannel activity may prevent the passage of regulatory factors (*e.g.*, ions and/or larger signaling molecules) through Cx30, thereby attenuating its inhibitory effect on ENaC.

It is well established that ENaC can be inhibited by intracellular Na^+^ due to so-called Na^+^ feedback inhibition (79, 80, 81, 82, 83). In the oocyte system, this mechanism is activated by intracellular Na^+^ concentrations above 30 to 40 mM and probably involves the PPPxY-motifs and Nedd4-2-dependent mechanisms (80, 81, 83, 84). In the present study, oocytes were incubated after cRNA injection in a solution with a relatively low Na^+^ concentration (approximately 14 mM), which we routinely use to minimize ENaC-mediated Na^+^ influx and subsequent Na^+^ feedback inhibition (63, 82, 85). Moreover, we demonstrated that the inhibitory effect of Cx30 was not affected by coexpressing catalytically inactive *Xenopus* xNedd4-CS. Thus, under our experimental conditions, it is unlikely that Cx30-mediated Na^+^ influx and Nedd4-2-dependent Na^+^ feedback inhibition contribute to the inhibitory effect of Cx30 on ENaC. However, a minor contribution cannot be ruled out and may play a role under conditions with enhanced Cx30-mediated Na^+^ influx.

It has been shown that Cx26, a close homologue of Cx30, is permeable for Ca^2+^ (86). Interestingly, in Cx30 expressing oocytes, reapplication of divalent cations after divalent cation removal regularly elicited a rapid and transient inward current peak (Figs. 1*A* and 3A), which preceded the inhibitory effect of divalent cations on Cx30-mediated inward currents. Such a peak response is a well-known phenomenon in oocytes and most likely caused by a transient stimulation of Ca^2+^-activated chloride channels (53, 64, 87). This suggests that Cx30 may not be fully blocked in the presence of physiological concentrations of divalent cations on the extracellular side, but may be slightly permeable for Ca^2+^ and therefore stimulate Ca^2+^ signaling pathways. Previous studies provide evidence that an increased intracellular Ca^2+^ concentration downregulates ENaC by complex mechanisms (79, 88, 89, 90, 91, 92, 93). However, as mentioned above, there was an inverse relationship between the extracellular Ca^2+^ concentration and the inhibitory effect of Cx30 on ENaC. Moreover, replacing extracellular Ca^2+^ with equimolar Ba^2+^ did not prevent the inhibitory effect of Cx30 on ENaC, but rather enhanced it. Thus, the inhibitory effect of Cx30 on ENaC does not require the presence of Ca^2+^ in the extracellular solution. Therefore, it is unlikely that Cx30-mediated Ca^2+^ influx is essential for causing ENaC inhibition. Nevertheless, we cannot exclude the possibility that Ba^2+^ may pass through Cx30 and stimulate intracellular signaling pathways, which are normally activated by Ca^2+^. Examples can be found in the literature that Ba^2+^ can substitute for Ca^2+^ in mediating biological responses (94, 95, 96).

Our data clearly demonstrate that Cx30 decreases ENaC surface expression by enhancing channel retrieval without having a significant effect on the total intracellular amount of ENaC, its single-channel properties, or proteolytic processing. Moreover, our findings indicate that the C-termini of β- and γENaC are critically important for the inhibitory effect of Cx30 on ENaC. It is generally accepted that C-termini of β- and γENaC play the most important role in regulating channel surface expression due to the presence of conserved PPPxY-motifs (4, 7, 15, 16). Naturally occurring mutations causing C-terminal truncations in β- or γENaC are known to cause Liddle syndrome—a severe form of salt-sensitive arterial hypertension (9, 97, 98). The PPPxY-motifs are binding sites for the ubiquitin ligase Nedd4-2, which ubiquitinates the N-termini of ENaC and promotes channel internalization and degradation (13, 15, 16, 17, 18, 19, 20, 21). As stated above, Nedd4-2-dependent ENaC retrieval from the cell surface is thought to play a major role in Na^+^ feedback inhibition (80, 83, 84). Moreover, it is critically involved in mediating ENaC inhibition by progesterone (99) and channel phosphorylation, *e.g.*, by ERK or CK-2 kinases (100, 101, 102). The present study indicates that the conserved prolines of the PPPxY-motifs and Nedd4-2-dependent mechanisms are not critically involved in mediating ENaC inhibition by Cx30. In contrast, we demonstrate that pharmacological inhibition of clathrin-mediated endocytosis by Pitstop-2 largely reduced ENaC inhibition by Cx30. Importantly, truncating the YxxΦ-motif in γ-ENaC or replacing leucine residues by negatively charged residues in the YxxΦ-motifs of γ-ENaC and/or β-ENaC not only increased baseline ENaC currents but also significantly reduced the inhibitory effect of Cx30 on ENaC. Furthermore, Cx30-mediated ENaC inhibition was slightly but significantly reduced by mutating the critical tyrosine residues, which are part of the PPPxY-motifs but also of the YxxΦ-motifs. Taken together, these findings suggest that coexpression of Cx30 stimulates YxxΦ-motif-dependent clathrin-mediated retrieval of ENaC from the cell surface. YxxΦ-motifs are well-known binding sites for μAP2 and are critically involved in clathrin-mediated endocytosis of various cell surface proteins including ionotropic GABA_A_ and P2X4 receptors (26, 28, 29). Our finding that the inhibitory effect of Cx30 was not affected by catalytically inactive *Xenopus* xNedd4-CS indicates that the stimulatory effect of Cx30 on clathrin-mediated endocytosis of ENaC is independent of Nedd4-2. This supports the previously proposed concept of two independent pathways of ENaC retrieval, *i.e.*, Nedd4-2-mediated and AP2-mediated (30). Interestingly, in a previous study it has been demonstrated that the disruption of the Nedd4-2-binding motifs dramatically reduced ENaC endocytosis, whereas mutating the YxxΦ-motif had no effect on the rate of ENaC retrieval (65). However, in the latter study, a threonine residue within the YxxΦ-motif of γENaC was mutated to alanine (γT629A). This mutation has been reported to disrupt ENaC precipitation with AP2 and to prevent ENaC stimulation by coexpression of dominant negative dynamin, which blocks endocytosis *via* clathrin-coated pits (30). Interestingly, this threonine residue precedes the critical leucine residue mutated in the current study, and its substitution by alanine may not fully abolish the function of the YxxΦ-motif. Moreover, different expression systems may account for some of the discrepant findings. For example, Nedd4-2 overexpression increased ENaC endocytosis in human embryonic kidney 293 cells but not in Fischer rat thyroid epithelial cells (65). Thus, it is tempting to speculate that an alternative usage of Nedd4-2-mediated or AP2-mediated ENaC retrieval may be physiologically important to regulate ENaC expression at the cell surface in a tissue-specific manner. In Liddle patients, C-terminal truncations of β- and γENaC not only disrupt the PPPxY-motif of the affected subunit but can also affect the YxxΦ-motifs. Therefore, both pathways involved in ENaC retrieval may contribute to the pathophysiology of Liddle syndrome.

The direct mechanistic link of how Cx30 ion channel function promotes clathrin-mediated ENaC endocytosis remains to be elucidated. In this context, additional studies are needed to identify the relevant ions and/or signaling molecules, which enter the cell and/or are released from the cell through Cx30 hemichannels and are responsible for stimulating the endocytic machinery. It is also still unclear whether Cx30 mediates its inhibitory effect on ENaC mainly in a paracrine/autocrine manner or whether Cx30 has to be coexpressed in the same cell to stimulate clathrin-mediated ENaC retrieval. Moreover, it will be important to determine the specificity of the effect of Cx30 on ENaC, because a stimulation of clathrin-mediated endocytosis by Cx30 may affect the retrieval of a wide range of membrane proteins. For example, we observed in pilot experiments that Cx30 coexpression significantly reduced ROMK-mediated currents in the oocyte expression system (*data not shown*). ROMK is a secretory K^+^ channel colocalized with ENaC in the apical membrane of CD principal cells and known to be internalized in a clathrin-dependent manner (103, 104). However, there is no indication for increased ROMK function in Cx30^−/−^ mice due to reduced clathrin-mediated ROMK retrieval. In particular, Cx30^−/−^ mice are not hypokalemic (34), which would be an expected consequence of an increased ROMK activity in the context of an increased ENaC activity enhancing the electrochemical driving force for renal potassium secretion through ROMK. Moreover, pressure-induced natriuresis was significantly blunted in Cx30^−/−^ mice, but there was no difference in pressure-induced kaliuresis in wild-type and Cx30^−/−^ mice (34). These findings suggest that Cx30 deficiency enhances ENaC activity without affecting ROMK activity, which indicates some degree of specificity of the functional interaction between Cx30 and ENaC *in vivo*. The precise localization of Cx30 in the luminal membrane of distal tubular epithelium varies in different species (33). Interestingly, in mice, Cx30 expression was found to be particularly strong in the apical membrane of intercalated cells of CDs (33). Therefore, Cx30 may also regulate ion channels expressed in intercalated cells, which remains to be investigated.

It will also be of interest to explore whether other connexins may affect clathrin-mediated endocytosis. Indeed, in preliminary experiments, we observed that coexpression of Cx37 also significantly reduced ENaC-mediated currents in the oocyte expression system (*data not shown*). However, only basolateral localization of Cx37 has been described in various nephron segments (105) and Cx37^−/−^ mice lack an obvious renal phenotype (35, 106). Thus, Cx30 is a more likely candidate than Cx37 to modulate ENaC function *in vivo*. Nevertheless, modulation of ion channel function by connexin hemichannels *via* stimulation of clathrin-mediated endocytosis may not be limited to ENaC and Cx30 but may represent a more general regulatory mechanism.

In conclusion, the *in vitro* findings of the present study reveal a novel mechanism of ENaC inhibition by Cx30. Absence of this inhibitory effect may contribute to increased ENaC activity and salt-sensitive hypertension in mice with Cx30 deficiency (33, 34, 35, 36, 37). Moreover, this study highlights the regulatory potential of modifying the rate of clathrin-mediated channel retrieval to adjust ENaC expression at the cell surface.

## Experimental procedures

### cDNA clones and antisense oligonucleotides

Full-length cDNAs for human α-, β-, and γENaC were kindly provided by H. Cuppens (Leuven, Belgium). Full-length cDNA encoding human connexin 30 (Cx30) was provided by Source BioScience UK Limited (I.M.A.G.E. Clone ID 5196769; (107)). Full-length cDNA for catalytically inactive *X. laevis* Nedd4-2 (xNedd4-CS; (73)) was kindly provided by O. Staub (Lausanne, Switzerland). cDNAs were subcloned into the pTLN vector (108). Linearized plasmids were used as templates for cRNA synthesis using T7 RNA polymerases (mMessage mMachine, Ambion). Truncations and single-point mutations in ENaC subunits as well as single-point mutation (M34A) and C-terminal insertion of V5-tag in Cx30 were generated using QuikChange Lightening site-directed mutagenesis kit (Agilent Technologies). Sequences were confirmed by sequence analysis (LGC Genomics). An antisense phosphothioate-oligoDNA against *X. laevis* Cx38 was synthesized by biomers.net GmbH.

### Chemicals

The sulfhydryl reagent MTSET was obtained from Biotium. Amiloride hydrochloride, connexin inhibitor carbenoxolone, clathrin inhibitor Pitstop-2, and α-chymotrypsin type II from bovine pancreas were purchased from Sigma-Aldrich.

### Isolation of oocytes and two-electrode voltage clamp experiments

Isolation of oocytes and two-electrode voltage clamp experiments were performed essentially as described previously (64, 68, 69, 71, 109). Defolliculated stage V to VI oocytes were obtained from ovarian lobes of adult female *X. laevis* in accordance with the principles of German legislation, with approval by the animal welfare officer for the University of Erlangen-Nürnberg and under the governance of the state veterinary health inspectorate (approval number Az. 55.2-2532-2-527). Animals were anesthetized in 0.2% MS222 (Sigma), and ovarian lobes were obtained by a small abdominal incision. Oocytes were injected with the same amount (0.1 ng unless stated otherwise) of cRNA per ENaC subunit (α, β and γ) per oocyte. Unless stated otherwise, 2 ng of human Cx30 cRNA was injected. The amount of *X. laevis* xNedd4-CS cRNA was 3 ng per oocyte. To suppress the expression and possible interference of endogenous connexin 38 (Cx38) hemichannels, an established approach (50, 51) was used, *i.e.*, 3 ng of the antisense phosphothioate-oligoDNA against Cx38 (AS Cx38) corresponding to nucleotides −5 to +25 of the coding region of Cx38 (5′-GCT TTA GTA ATT CCC ATC CTG CCA TGT TTC-3′) was routinely coinjected into the oocytes together with ENaC and/or Cx30 cRNA. To prevent Na^+^ overloading, which would reduce ENaC cell surface expression, injected oocytes were maintained in a low-sodium ND9 solution (in mM: 9 NaCl, 2 KCl, 87 N-methyl-D-glutamine-Cl, 1.8 CaCl_2_, 1 MgCl_2_, 5 HEPES, pH 7.4 adjusted with Tris) supplemented with 100 units/ml sodium penicillin and 100 μg/ml streptomycin sulphate. Unless stated otherwise, concentration of CaCl_2_ in ND9 incubation solution was 1.8 mM. Penicillin and streptomycin were omitted from ND9 when 1.8 mM CaCl_2_ was replaced by 1.8 mM BaCl_2_ to avoid precipitation of insoluble salts. Oocytes were studied 48 h after cRNA and AS Cx38 injection. Bath solution exchanges with a gravity-fed system were controlled by a magnetic valve system (ALA BPS-8) in combination with a TIB14 interface (HEKA). An individual oocyte was placed in an experimental chamber with a narrow flow channel (length: 45 mm; height: 3 mm; width: 3 mm) with a U-shaped cross section of ∼8 mm^2^. The oocyte was positioned in the experimental chamber close to the site of solution inflow and was held in place by the impaling microelectrodes. To achieve rapid and reproducible solution exchanges at the oocyte, the perfusion rate was carefully adjusted for each experimental solution to ∼10 ml/min, resulting in a flow velocity of ∼20 mm/s. The flow channel drained into a reservoir (2 cm × 1 cm) from which the solution was continuously removed *via* a suction tube. The suction tube was adjusted to maintain the fluid level in the flow channel at ∼2 mm. Oocytes were clamped at a holding potential of −60 mV using an OC-725C amplifier (Warner Instruments) connected by a LIH-1600 (HEKA) to a personal computer. Pulse 8.78 software (HEKA) was used for data acquisition. ENaC-mediated whole-cell currents (ΔI_Ami_) were determined by washing out amiloride (2 μM) with amiloride-free bath solution and subtracting the whole-cell currents measured in the presence of amiloride from the corresponding whole-cell currents recorded in the absence of amiloride. ND96 solution was used as standard bath solution (in mM: 96 NaCl, 2 KCl, 1.8 CaCl_2_, 1 MgCl_2_, 5 HEPES, pH 7.4 adjusted with Tris). Activity of Cx30 (or Cx38) was revealed by measuring whole-cell currents (ΔI_Ca_^_2+_^_Mg_^_2+_^_-Removal_) elicited by extracellular removal of divalent cations (Ca^2+^, Mg^2+^). To obtain a Ca^2+^ and Mg^2+^ -free bath solution, 2 mM EDTA was added instead of 1.8 mM CaCl_2_ and 1 MgCl_2_ (in mM: 96 NaCl, 2 KCl, 2 EDTA, 5 HEPES, pH 7.4 adjusted with Tris). NaCl (95 mM) was replaced by NMDG-Cl to obtain a low-sodium NMDG-Cl bath solution (in mM: 95 NMDG-Cl, 1 NaCl, 2 KCl, 1.8 CaCl_2_, 1 MgCl_2_, 5 HEPES, 7.4 pH adjusted with Tris). The residual activity of Cx30 in the presence of divalent cations (1.8 mM Ca^2+^, 1 mM Mg^2+^) was assessed using the unspecific connexin inhibitor carbenoxolone (1 mM).

The cation permeability ratio, P_NMDG_/P_Na_, was calculated using the following equation (110):

$$P_{NMDG}/P_{Na}=e^{\Delta E_{rev}F/RT}$$

where $\Delta E_{rev}\;$ is the shift of the reversal potential $\left(E_{rev}\right)$ caused by replacing Na^+^ in the bath solution with NMDG^+^, F is Faraday’s constant, R is the universal gas constant, and T is the absolute temperature.

ΔI_Retrieval_ (see Fig. 9) was calculated according to the following equation:

ΔIRetrieval = ΔIAmi(0min)−(ΔIAmi(70min)−ΔIInsertion)

ΔI_Insertion_, *i.e.*, the component of ΔI_Ami (70 min)_ mediated by MTSET responsive channels newly inserted into the plasma membrane within 70 min, was calculated from the ΔI_Ami_ increase due to the second application of MTSET (ΔI_2nd MTSET_) according to the following equation, which takes into consideration the increase in P_o_ due to MTSET:

$$\Delta {\text{I}}_{\text{Insertion}}=\;\Delta {\text{I}}_{2\text{nd}\;\text{MTSET}}\;{\text{P}}_{\text{o}\;\left(\text{initial}\right)}/\left(1-{\text{P}}_{\text{o}\;\left(\text{initial}\right)}\right)$$

where P_o (initial)_ was calculated as follows:

$${\text{P}}_{\text{o}\;\left(\text{initial}\right)}=\Delta {\text{I}}_{\text{Ami}\;\left(\text{before}\;1\text{st}\;\text{MTSET}\right)}/\Delta {\text{I}}_{\text{Ami}\;\left(\text{0}\;\text{min}\right)}$$

This calculation is based on the assumption that average P_o_ of ENaC before the first exposure to MTSET is similar to that of newly inserted channels before the second MTSET application. Moreover, it was assumed that when MTSET was applied for a second time, it only increased P_o_ of newly inserted channels to ∼1 and had no effect on channels covalently modified by the first MTSET application.

### Surface expression assay and western blot analysis

Surface ENaC expression was determined using a chemiluminescence assay as described previously (62, 63, 64). For these experiments, cRNAs (0.6 ng per oocyte per subunit) encoding wild-type α- and γ-ENaC were coinjected with cRNA encoding β-ENaC with a FLAG tag inserted into its extracellular loop. Mouse monoclonal anti-FLAG M2 antibody (Sigma-Aldrich) as primary antibody and peroxidase-conjugated sheep anti-mouse IgG (Chemicon) as secondary antibody were used. Individual oocytes were placed in a white U-bottom 96-well plate, and 50 μl of SuperSignal ELISA femto maximum sensitivity substrate (Thermo Scientific) was added to each oocyte. Chemiluminescence was quantified with a Tecan GENios microplate reader (TECAN). Results are given in relative light units (RLU).

To separate cell surface expressed Cx30 from intracellular Cx30, cell surface proteins were labeled with biotin essentially as described previously (64, 102). Oocytes (30/group) were incubated for 15 min in a biotinylation buffer (2.5 ml; pH 9.5) containing 10 mM triethanolamine, 150 mM NaCl, 2 mM CaCl_2_, and 1 mg ml^−1^ EZ-linked sulfo-NHS-SS-Biotin (Pierce). After biotinylation was completed, oocytes were lysed in homogenization buffer (1 ml; pH 7.9) containing 83 mM NaCl, 10 mM HEPES, 1 mM MgCl_2_, supplemented with 0.5% Triton X-100, 0.5% Igepal CA-630 (Sigma-Aldrich) and a protease inhibitor cocktail (complete EDTA-free, Roche Diagnostics). The lysate was centrifuged for 10 min at 1000*g* to remove cell debris and yolk proteins. The protein concentration of the resulting cell lysate was determined with a BCA-assay (Pierce). By adding 100 μl of immunopure immobilized NeutrAvidin Agarose beads (Pierce) to ∼850 μl of this lysate, biotinylated proteins were precipitated on a rotating wheel at 4 °C overnight. After subsequent centrifugation at 1000*g* for 3 min, beads (with bound cell surface proteins) and supernatant (intracellular proteins) were boiled for 5 min at 95 °C with sodium dodecyl sulphate-polyacrylamide gel electrophoresis (SDS-PAGE) sample buffer (Rotiload 1, Roth). Cell surface proteins were separated from beads by centrifugation at 16,000*g* for 3 min. The resulting preparation of cell surface proteins (30 μl of the total volume of ∼90 μl) and intracellular proteins (30 μg of total protein) was loaded on the 10% SDS-PAGE and transferred to polyvinylidene fluoride (PVDF) membranes by semidry electroblotting. V5-tagged Cx30 was detected using mouse monoclonal anti-V5 antibody (Invitrogen) at a dilution of 1:1000 and a secondary horseradish-peroxidase-labeled goat anti-mouse antibody (Abcam) at a dilution of 1:50,000. To validate separation of cell surface proteins from intracellular proteins by biotinylation, blots were stripped and reprobed using a polyclonal rabbit anti-β-actin antiserum (Sigma-Aldrich) at a dilution of 1:5000.

To estimate total ENaC expression in whole-cell lysates, 25 oocytes per group were homogenized with a 27-gauge needle in 1 ml of homogenization buffer (in mM: 10 HEPES, 83 NaCl, 1 MgCl_2_, pH 7.9) supplemented with protease inhibitor mixture (“Complete EDTA-free” protease inhibitor mixture tablets, Roche Diagnostics). The lysates were centrifuged for 10 min at 1000*g* and supernatants were incubated with 1% Triton X-100 on ice for 40 min. All samples were boiled for 5 min at 95 °C and subjected to 10% SDS-PAGE using 40 μg of total protein per lane. After separation, proteins were transferred to PVDF membranes by semidry electroblotting and probed with a subunit-specific antibodies against human α- (1:5000), β- (1:10,000), or γ-ENaC (1:5000) obtained from Pineda Antibody Service as described previously (64, 70, 71). Horseradish-peroxidase-labeled secondary goat anti-rabbit antibodies were purchased from Santa Cruz Biotechnology and used at a dilution of 1:50,000. Chemiluminescence signals were detected using Super Signal West Femto (Thermo Scientific). Densitometry was performed using ImageJ (National Institutes of Health, Bethesda, MD). ATX Ponceau S (Fluka) membrane staining was used to control protein loading.

### Single-channel recordings in outside-out patches

Single-channel recordings in outside-out membrane patches of αβγENaC expressing oocytes were performed 48 h after cRNA injection essentially as described previously (64, 68, 109) using conventional patch clamp technique. Patch pipettes were pulled from borosilicate glass capillaries and had a tip diameter of about 1 to 1.5 μm after fire polishing. Pipettes were filled with K-gluconate pipette solution (in mM: 90 K-gluconate, 5 NaCl, 2 Mg-ATP, 2 EGTA, and 10 HEPES, pH 7.2 with Tris). Modified ND96 solution was used as bath solution (in mM: 96 NaCl, 4 KCl, 1 CaCl_2_, 1 MgCl_2_, and 10 HEPES, pH 7.4 adjusted with Tris). Seals were routinely formed in low-sodium NMDG-Cl bath solution in which NaCl (95 mM) was replaced by NMDG-Cl (95 mM). In this bath solution, the pipette resistance averaged about 7 MΩ. After seal formation, the NMDG-Cl solution was switched to a standard NaCl bath solution. For continuous current recordings, the holding potential was set to −70 mV using an EPC9 amplifier (HEKA). Using a 3 M KCl flowing boundary electrode, the liquid junction (LJ) potential occurring at the pipette/NaCl bath junction was measured to be 12 mV (bath positive) (111). Thus, at a holding potential of −70 mV, the effective *trans*-patch potential was −82 mV. This value is close to the calculated equilibrium potential of Cl^−^ (E_Cl_^−^ =−77.4 mV) and K^+^ (E_K_^+^ =−79.4 mV) under our experimental conditions. Experiments were performed at room temperature. To change from one bath solution to another, a conventional gravity-fed system controlled by a magnetic valve system (ALA BPS-8) was used in combination with a TIB14 interface (HEKA). Pulse 8.78 software (HEKA) was used for data acquisition. Single-channel current data were initially filtered at 1.25 kHz and sampled at 5 kHz. The current traces were digitally refiltered at 250 Hz to resolve the single-channel current amplitude (i) and channel activity. The latter was derived from binned amplitude histograms as the product NP_o_ (64, 109, 111, 112). The current level at which all channels are closed was determined in the presence of 2 μM amiloride. The apparent number of active channels (apparent N) in a patch was determined by visual inspection of the current traces. Apparent channel open probability P_o(app)_ was calculated by dividing NP_o_ by apparent N for recordings with no more than three active channels in the patch (1 ≤ N ≤ 3). Single-channel data were analyzed using the program Nest-o-Patch written by Dr V. Nesterov (Institut für Zelluläre und Molekulare Physiologie, Friedrich-Alexander-Universität Erlangen-Nürnberg, Erlangen, Germany).

### Statistical analysis

Data are presented as mean ± SEM. Normal distribution of data was assessed using D’Agostino–Pearson omnibus test. Statistical significance was assessed by an appropriate parametric test: ANOVA (with Bonferroni post hoc test) or Student’s *t*-test or by a nonparametric test: Kruskal–Wallis (with Dunn’s post hoc test), Mann–Whitney, or Wilcoxon signed-rank test as indicated. N indicates the number of different batches of oocytes, and *n* indicates the number of individual oocytes studied per experimental group. Statistical analysis was performed using Graph Pad Prism 5.04.

## Data availability

All data are contained within the article.

## Conflict of interest

The authors declare that they have no conflicts of interest with the contents of this article.
